# Global, regional, and national burden of 10 digestive diseases in 204 countries and territories from 1990 to 2019

**DOI:** 10.3389/fpubh.2023.1061453

**Published:** 2023-03-28

**Authors:** Rui Wang, Zhaoqi Li, Shaojun Liu, Decai Zhang

**Affiliations:** ^1^Department of Gastroenterology, The Third Xiangya Hospital of Central South University, Changsha, Hunan, China; ^2^Hunan Key Laboratory of Non-resolving Inflammation and Cancer, Changsha, Hunan, China

**Keywords:** digestive diseases, global burden of disease, epidemiology, disability-adjusted life-years, estimated annual percentage change

## Abstract

**Background:**

Digestive diseases are very common worldwide and account for considerable health care use and expenditures. However, there are no global population-based estimates of the disease burden and temporal trend of digestive diseases.

**Methods:**

Annual case numbers, age-standardized rates of prevalence, incidence, death, and disability-adjusted life-years (DALYs), and their estimated annual percentage changes (EAPCs) for digestive diseases between 1990 and 2019 were derived from the Global Burden of Disease, Injuries, and Risk Factors Study (GBD) 2019. The association between digestive disease burden and the sociodemographic index (SDI) was investigated. We also calculated DALYs attributable to risk factors that had evidence of causation with digestive diseases.

**Results:**

Globally, in 2019, there were 88.99 million DALYs due to digestive diseases (3.51% of global DALYs). Digestive diseases were the 13th leading cause of DALYs globally in 2019. Global digestive disease DALYs were highest in the middle SDI quintile and in South Asia and were higher in males than females in 2019. Cirrhosis and other chronic liver diseases constituted the highest proportion of categorized digestive disease DALY burdens globally. From 1990 to 2019, the global age-standardized DALY rate of digestive diseases decreased from 1570.35 in 1990 to 1096.99 in 2019 per 1,00,000 population, with the EAPC being −1.32 (95% confidence interval [CI] −1.36 to −1.27). In 2019, the largest contributor to digestive disease DALYs at the global level, for both sexes, was alcohol use.

**Conclusion:**

The results of this systematic analysis suggest that the global burden of digestive diseases is substantial and varies markedly according to age, sex, SDI, and geographical region. These results provide comprehensive and comparable estimates that can potentially inform efforts toward digestive disease control worldwide.

## Introduction

Digestive diseases are highly prevalent worldwide, cause considerable distress, and can be fatal. Digestive diseases account for substantial health care utilization and spending (1, 2). Many of these diseases also affect patients' quality of life and productivity (3). In the United States, digestive diseases affect more than 40 million people and account for millions of clinical visits annually, with health care expenditures totaling $119.6 billion in 2018 (1). Similarly, in developing countries, the burden of digestive diseases has been increasing (4). Given the serious economic impact and significant social cost, epidemiologic data estimating digestive disease burden are critical for public health research, education and resource allocation.

The epidemiology of digestive diseases has changed over the past decades (5–7). Studies examining temporal trends in the burden and healthcare expenditure of digestive diseases have been conducted, but most have focused on the United States and Europe (1, 2, 8, 9). However, limited data is available for low to middle-income countries. Furthermore, no comprehensive population-based estimates of the global burden of digestive diseases exist to date. In addition, epidemiological data for some digestive diseases, including vascular intestinal disorders and intestinal obstructions, remain outdated or non-existent. The Global Burden of Diseases, Injuries, and Risk Factors Study (GBD) 2019 is a systematic worldwide effort that includes the assessment of the burden of digestive diseases (10), which provides a unique opportunity to understand the state of digestive diseases.

Our study aimed to present a comprehensive and up-to-date evaluation of the global burden of non-malignant digestive diseases. We utilized data from the GBD 2019 study to determine the global, regional and national burdens of digestive diseases across 204 countries and territories from 1990 to 2019 by age, gender and social-development index (SDI). The availability of burden of disease estimates for digestive diseases would provide further resources for better understanding the impacts of digestive diseases on population health and in determining the best strategies for reducing total disease burden.

## Methods

### Overview

The GBD 2019 study produced estimates for 369 diseases and injuries and 87 risk factors in 204 countries and territories from 1990 to 2019 using a unified and comparable method. The study integrated all available data sources identified through a literature review and research collaborations, including microdata from registry and cohort studies, scientific reports of cohorts and registries, health system administrative data, and population surveys (11). A detailed description of the methodology for collecting and processing these data and informing the results in GBD 2019 has been described in previous publications (10–13). In brief, after data collection, a Bayesian meta-regression tool (DisMod-MR 2.1) was used to model and derive estimates of the burden under several conditions (11). The model included all of the above available information for each disease and applied a correcting process for known bias to derive country-specific estimates of prevalence and of the burden of diseases, which have been described in previous studies (10). The 95% uncertainty intervals (UIs) reported for each estimate used 1,000 draws from the posterior distribution of models, reported as the 2.5th and 97.5th values of the distribution (11). As a result, GBD 2019 provides comprehensive and systematic assessments of age- and sex-specific incidence, prevalence, mortality, years of life lost (YLLs), years lived with disability (YLDs), and disability-adjusted life-years (DALYs) for 369 diseases and injuries. Detailed methods of GBD 2019 are reported in the Supplementary methods and summarized here.

### Case definitions

The GBD 2019 cause list is organized as a four-level hierarchy, with each level composed of causes of death that are mutually exclusive and collectively exhaustive (11). The four levels within the cause hierarchy and the position of each digestive disease within the hierarchy are presented in Supplementary Figure 1. The digestive diseases included in GBD 2019 were cirrhosis and other chronic liver diseases (five causes due to hepatitis B, hepatitis C, alcohol-related liver disease, non-alcoholic steatohepatitis, and other causes); upper digestive system diseases (peptic ulcer disease, gastritis and duodenitis, and gastroesophageal reflux disease); appendicitis; paralytic ileus and intestinal obstruction; inguinal, femoral, and abdominal hernia; inflammatory bowel disease (a combined estimate of all subtypes); vascular intestinal disorders (a combined estimate of all subtypes); gallbladder and biliary diseases (a combined estimate of all subtypes); pancreatitis (a combined estimate of all subtypes); and other digestive diseases (an aggregate group of other diseases of the digestive system). To allow for comparability in measurement, case definitions predominantly adhered to the 10th revisions of the International Classification of Diseases (ICD-10) (Supplementary Table 1).

### Data source

We extracted yearly crude and age-standardized estimates of various measures of the burden of digestive disease from 1990 to 2019 and the respective 95% UIs from the GBD database *via* the Global Health Data Exchange (GHDx) query tool (http://ghdx.healthdata.org/gbd-results-tool). The variables obtained from the database included incident cases, prevalent cases, deaths, YLLs, YLDs, DALYs, and their corresponding age-standardized rates (ASRs) at the global, regional, and national levels. These data were stratified by age (0–4, every 5-year age group up to 95 years and 95 years and older), calendar year (1990-2019), region, and country (or territory). Geographically, the world was divided into seven super-regions and 21 regions. Moreover, the 204 countries and territories were categorized into five groups in terms of their sociodemographic index (SDI): high, high-middle, middle, low-middle, and low SDI quintiles (11).

### Attributable risk factors

Smoking, alcohol use, drug use, and high body-mass index were the four risk factors for digestive diseases available in GBD database and included in our analysis. We assessed the number contribution of these factors to digestive diseases DALYs in 2019 *via* GBD results tool, and then calculate the percentage contribution. The total number of input data sources used for the exposure of different risk factors in GBD 2019 is detailed elsewhere (13), of which we present the number of input data sources for the three risk factors related to digestive diseases.

### Statistical analysis

Estimated annual percentage changes (EAPCs) were computed to depict the temporal trend in various ASRs of digestive disease burden, which were calculated using a regression model. The whole process includes two steps. Step 1: Linear regression of 30 years' data; that is, y = α+βx+ε, where y = ln (rate), x = calendar year, and ε = error term. In this formula, β represents the positive or negative ASR trends. Step 2: Calculation of linear regression parameters; that is, EAPC = 100 × [exp (β)−1] (14). A 95% confidence interval (CI) for each quantity was calculated for the analyses. When the EAPC value and its 95% CI are greater than zero, the ASR is upwards. Conversely, when both values are less than zero, the ASR is downwards. Otherwise, the ASR was regarded as stable over time. All statistical analyses and visualizations were conducted using the R statistical software program (version 4.1.0). A *P*-value < 0.05 was considered statistically significant.

Because the study was based on a publicly available dataset, this study was exempted by the ethics committee of the Third Xiangya Hospital of Central South University. This study followed the guidelines of a cross-sectional study described in the Guidelines for Accurate and Transparent Health Estimates Reporting (GATHER) (15).

## Results

**Figure 1:**
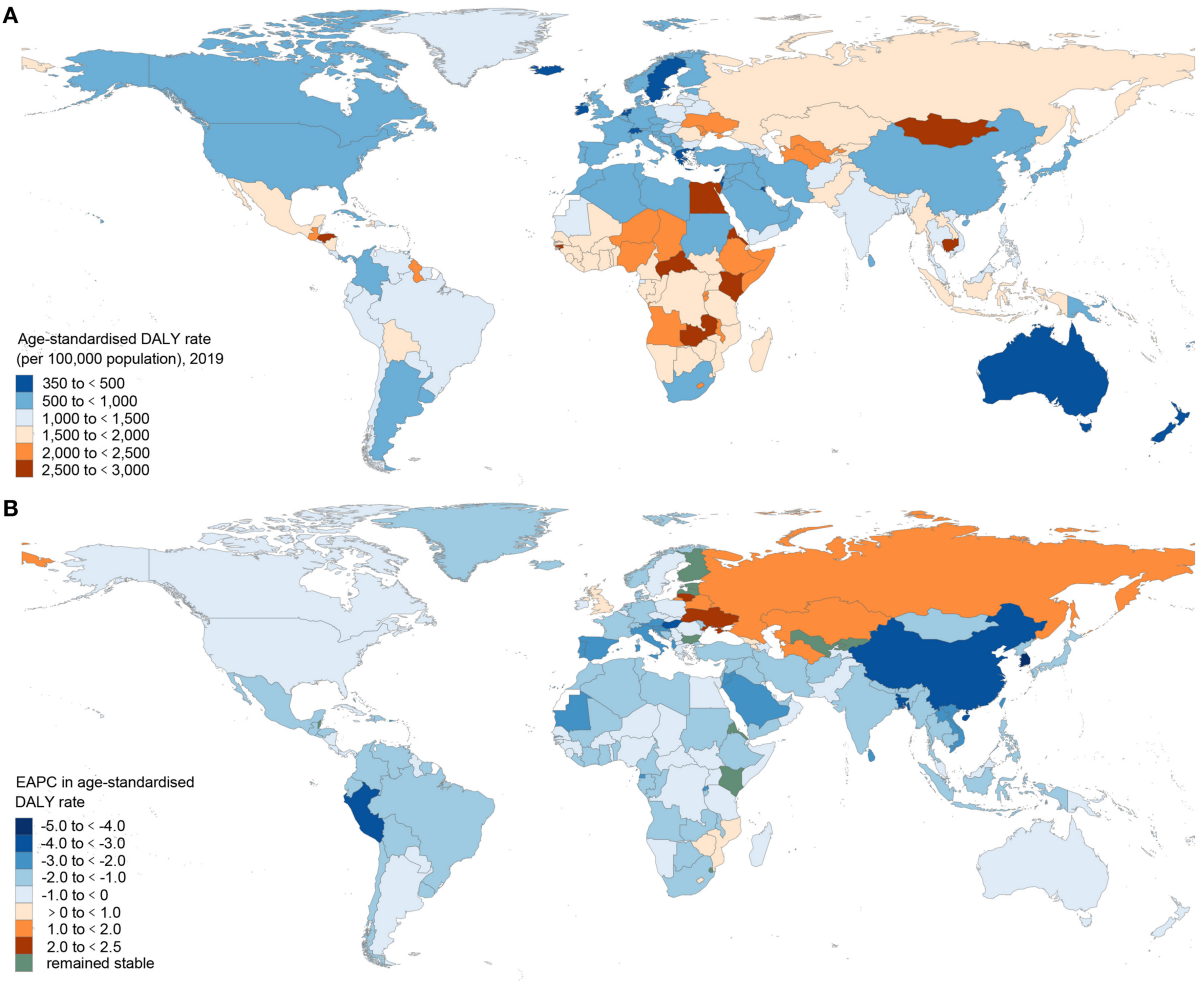
The global DALY burden of digestive diseases in 204 countries and territories. **(A)** The age-standardized DALY rate (per 100,000 population) of digestive diseases in 2019. **(B)** The EAPC of the age-standardized DALY rate for digestive diseases between 1990 and 2019. DALY, disability-adjusted life-year; EAPC, estimated annual percentage change.

Digestive diseases accounted for 2276.27 million (95% UI 2151.23–2398.06) estimated prevalent cases, 2.56 million (2.39–2.72) deaths, and 88.99 million (81.41–97.58) DALYs globally in 2019 (Table 1), which increased respectively by 67.87%, 37.85%, and 23.47% from 1990. Meanwhile, the global incident cases of digestive diseases were 443.53 million (405.58–484.42) in 2019, increasing from 254.25 million (231.57 to 277.83) in 1990 (Supplementary Table 2). A substantial portion of the global burden of digestive diseases exists in middle-, low-middle- and high-middle-SDI countries (72.78% of the global digestive disease total DALYs; Table 1) and countries that are concentrated in Africa, the Middle East, and Central Asia (Figure 1A and Supplementary Table 3). In 2019, the highest age-standardized DALY rates of digestive diseases were observed in Cambodia, Egypt, Central African Republic, Mongolia, and Honduras. By contrast, the lowest age-standardized DALY rates of digestive diseases were found in Iceland, Singapore, Switzerland, Malta, and Netherlands (Supplementary Table 3). From 1990 to 2019, the global age-standardized DALY rate of digestive diseases decreased from 1570.35 (1466.86 to 1677.67) per 100,000 population in 1990 to 1096.99 (1002.19 to 1202.93) in 2019, with an EAPC of −1.32 (95% CI −1.36 to −1.27) (Supplementary Table 4). Most of the countries or territories (89.71%) experienced a decrease in the age-standardized DALY rate, with the largest decrease in the Republic of Korea [EAPC = −4.62 (95% CI −4.94 to −4.30)]. In contrast, Ukraine showed the largest increase in the age-standardized DALY rate [EAPC = 2.30 (1.73 to 2.88)] (Figure 1B).

**Table 1:** Prevalent cases, mortalities, disability-adjusted life-years (DALYs) and their age-standardized rates due to digestive diseases in 2019 for both sexes.

	**Prevalent cases, in millions (95% UI)**	**Age–standardized prevalence rate per 100,000 population (95% UI)**	**Mortalities, in millions (95% UI)**	**Age–standardized mortality rate per 100,000 population (95% UI)**	**DALYs, in millions (95% UI)**	**Age–standardized DALY rate per 100,000 population (95% UI)**
**Global**	2276.27 (2151.23–2398.06)	27911.70 (26407.85–29378.01)	2.56 (2.39–2.72)	32.07 (29.87–34.05)	88.99 (81.41–97.58)	1096.99 (1002.19–1202.93)
**SDI quintile**						
High	278.40 (263.46–293.06)	21282.97 (20020.62–22472.05)	0.41 (0.37–0.43)	21.02 (19.33–22.03)	10.52 (9.60–11.53)	687.51 (625.27–762.32)
High–middle	480.05 (454.18–505.89)	27071.26 (25530.65–28540.60)	0.47 (0.43–0.49)	23.92 (22.20–25.42)	15.45 (14.17–17.13)	831.63 (761.22–925.25)
Middle	773.78 (731.60–817.92)	29603.29 (28029.84–31220.53)	0.73 (0.67–0.79)	31.42 (28.66–34.35)	25.15 (22.83–27.80)	991.00 (900.15–1092.85)
Low–middle	487.49 (460.16–513.93)	29473.92 (27910.86–31020.02)	0.63 (0.58–0.69)	46.21 (42.50–50.41)	24.13 (21.78–26.58)	1537.78 (1392.98–1686.63)
Low	255.23 (239.72–269.77)	30220.37 (28627.20–31762.76)	0.33 (0.29–0.37)	58.46 (52.22–65.25)	13.69 (11.92–15.66)	1813.89 (1599.27–2044.58)
**Disorders**						
Cirrhosis and other chronic liver diseases	1690.96 (1560.88–1845.46)	20710.05 (19127.33–22589.38)	1.47 (1.37–1.58)	18.00 (16.80–19.31)	46.19 (43.03–49.55)	560.43 (521.86–602.02)
Upper digestive system diseases	780.59 (694.90–863.84)	9539.88 (8491.56–10542.84)	0.27 (0.25–0.30)	3.48 (3.19–3.80)	14.87 (11.34–20.18)	182.81 (139.36–247.92)
Gallbladder and biliary diseases	193.49 (166.63–229.38)	2350.78 (2029.59–2778.69)	0.12 (0.11–0.14)	1.65 (1.41–1.84)	6.35 (4.87–8.25)	78.25 (60.29–101.40)
Inguinal, femoral, and abdominal hernia	32.53 (27.71–37.79)	407.93 (348.07–474.56)	0.05 (0.04–0.05)	0.63 (0.55–0.71)	3.35 (2.61–4.25)	42.75 (33.36–53.99)
Inflammatory bowel disease	4.90 (4.35–5.50)	59.25 (52.78–66.47)	0.04 (0.03–0.04)	0.54 (0.46–0.59)	1.62 (1.36–1.92)	20.15 (16.86–23.71)
Pancreatitis	2.41 (2.16–2.64)	29.28 (26.26–32.03)	0.12 (0.10–0.13)	1.43 (1.30–1.59)	3.64 (3.28–4.03)	44.40 (40.05–49.08)
Appendicitis	0.67 (0.54–0.85)	8.71 (6.88–11.01)	0.03 (0.03–0.04)	0.43 (0.35–0.48)	1.50 (1.24–1.71)	19.35 (15.92–21.98)
Paralytic ileus and intestinal obstruction	0.55 (0.53–0.57)	7.43 (7.10–7.73)	0.24 (0.20–0.27)	3.14 (2.62–3.51)	7.08 (5.65–8.25)	93.46 (74.42–109.76)
Vascular intestinal disorders	0.18 (0.16–0.20)	2.21 (1.98–2.48)	0.11 (0.10–0.12)	1.40 (1.24–1.53)	1.87 (1.72–2.03)	23.49 (21.58–25.59)
Other digestive diseases	NA	NA	0.10 (0.08–0.12)	1.38 (1.12–1.57)	2.50 (2.12–2.80)	31.89 (27.07–35.67)

NA, not available; SDI, Socio-demographic Index; UI, uncertainty interval.

**Figure 2:**
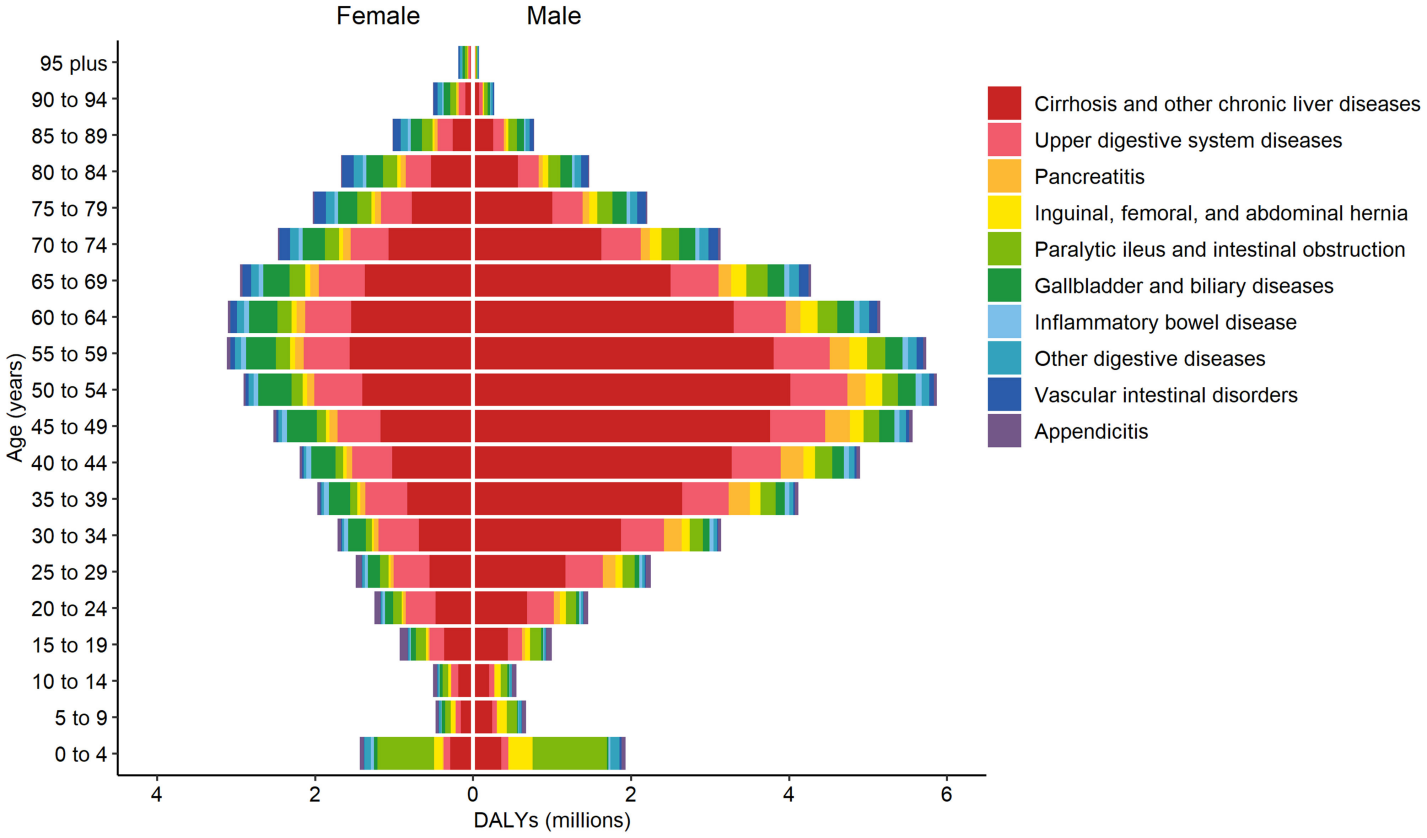
Global DALYs by digestive diseases, sex, and age, 2019. DALYs, disability-adjusted life-years.

Of the digestive disease age groups, the 50- to 59-year-old age group had the greatest contribution to digestive disease DALYs [8.85 million (95% UI 8.16–9.63), or 9.95% of the global all-age-years digestive disease absolute DALY burden; Figure 2]. Cirrhosis and other chronic liver diseases constituted the highest proportion of categorized digestive disease DALY burden globally, followed by upper digestive system diseases, with 51.9% of all digestive disease DALYs globally attributable to cirrhosis and other chronic liver diseases and 16.7% attributable to upper digestive system diseases (Supplementary Figure 2). Cirrhosis and other chronic liver diseases contributed to the greatest proportional categorized DALY burden globally in all age groups, except for the 0–4 year age group. In the 0–4 year age group, paralytic ileus and intestinal obstruction contributed the highest proportional DALY burden categorized (49.3%).

Globally, the number of DALYs for digestive diseases was higher in males [54.50 million (95% UI 49.90–59.47)] than females [34.49 million (30.95–38.79)] in 2019 (Figure 2). Cirrhosis and other chronic liver diseases; inguinal, femoral, and abdominal hernia; and pancreatitis were more common in males than females. Gallbladder and biliary diseases, appendicitis, and vascular intestinal disorders were more common in females.

**Figure 3:**
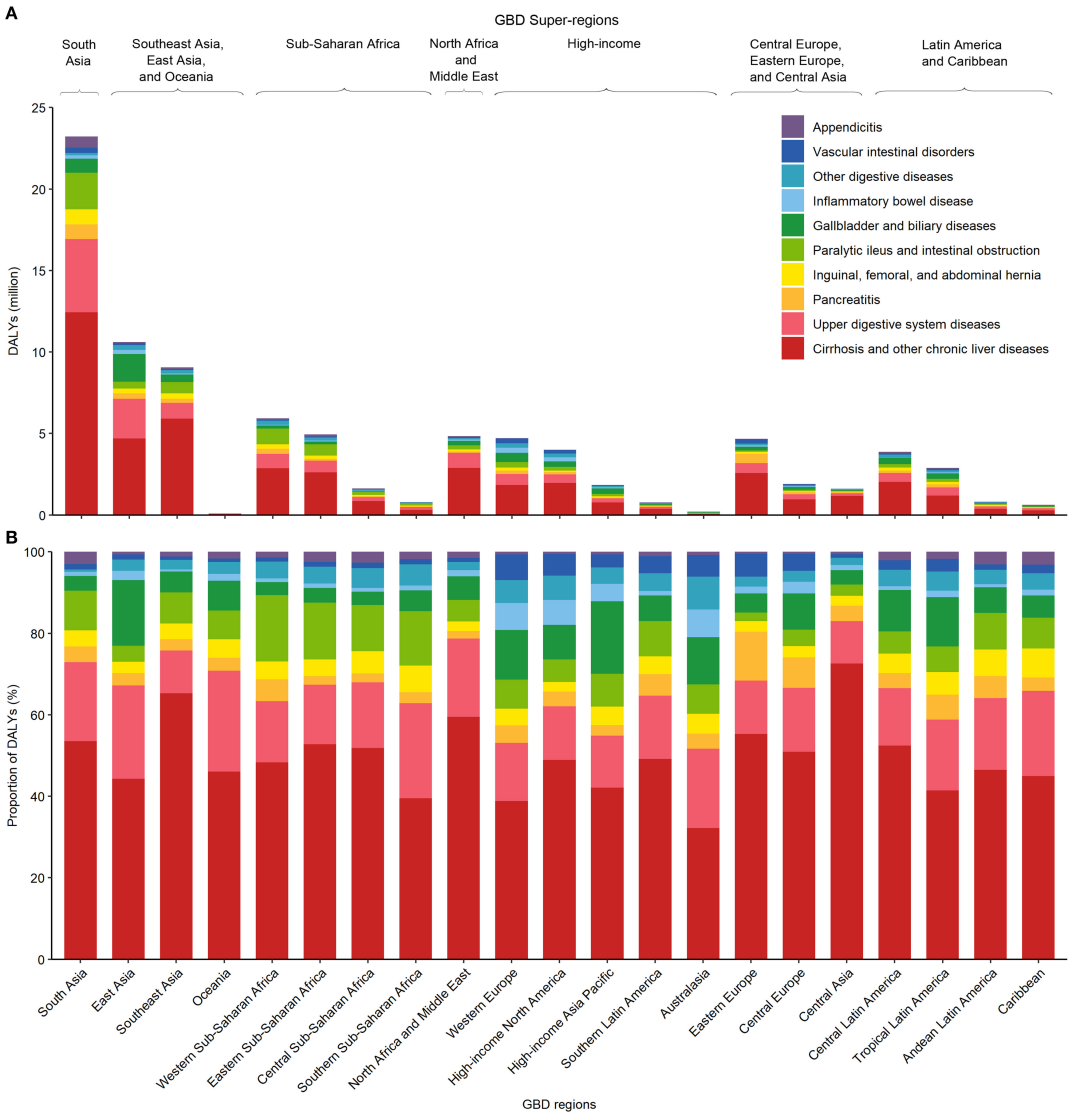
The absolute **(A)** and proportional **(B)** DALYs due to various digestive diseases by GBD world region, both sexes combined, 2019. DALY, disability-adjusted life-year; GBD, Global Burden of Disease, Injuries, and Risk Factors Study.

When assessed by GBD regions, there was substantial variability in the absolute and proportional DALY burden of digestive diseases by disease type (Figure 3 and Supplementary Table 5). Estimates of the proportion of digestive disease DALYs comprising cirrhosis and other chronic liver diseases and upper digestive system diseases, the most common digestive disease types, both varied between GBD regions by up to 2.25 and 2.38 times, respectively. The greatest proportional burden of cirrhosis and other chronic liver diseases was in Central Asia (72.60% of all digestive diseases) and Southeast Asia (65.27% of all digestive diseases), whereas the greatest absolute burden was in South Asia [12.44 million (95% UI 10.95–14.41) DALYs] and Southeast Asia [5.92 million (5.21–6.64) DALYs]. The greatest proportional burden of upper digestive system diseases was in Oceania (24.78% of all digestive diseases) and southern sub-Saharan Africa (23.38% of all digestive diseases), whereas the greatest absolute burden was in South Asia [4.49 million (3.40–6.12) DALYs] and East Asia [2.43 million (1.93–3.16) DALYs].

Globally, digestive diseases were the 15th leading cause of DALYs in 1990 and the 13th leading cause of DALYs in 2019. At the disorder level, cirrhosis and other chronic liver diseases were ranked 16th among the top 20 leading causes of DALYs in 2019 (Supplementary Figure 3). The rankings of digestive diseases differed by sex and age (Supplementary Figures 4, 5).

**Figure 4:**
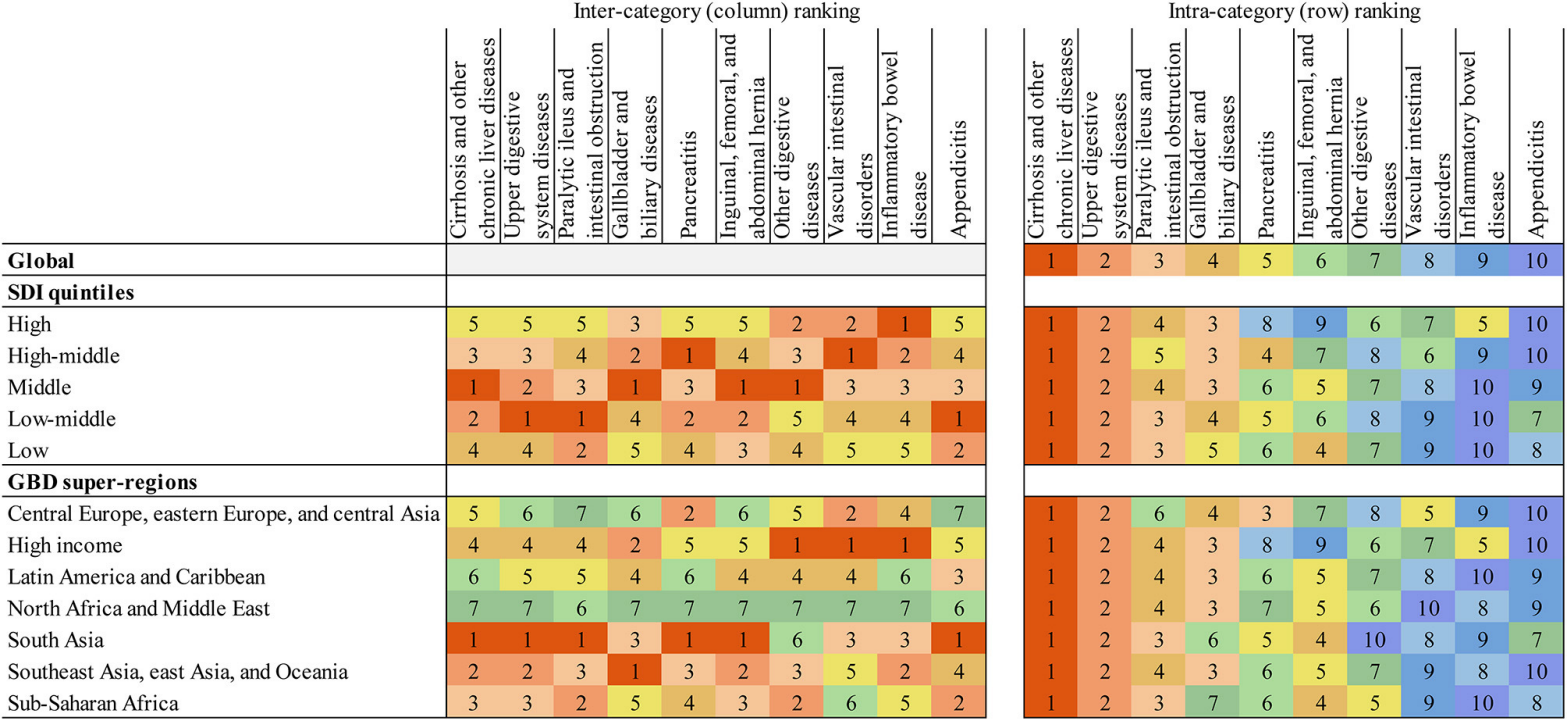
Digestive diseases ranked by numbers of DALYs for both sexes combined, 2019. Intercategory ranking refers to ranking vertically (ranking between the SDI quintiles and GBD super-regions). Intracategory ranking refers to ranking horizontally (ranking within each SDI quintile and GBD super-region). Number ranking is assigned by total absolute DALYs, with 1 representing the highest rank and greatest absolute DALY burden. DALYs, disability-adjusted life-years; GBD, Global Burden of Disease, Injuries, and Risk Factors Study; SDI, Sociodemographic Index.

Rankings of the relative burden of digestive diseases are shown in Figure 4, expressed in absolute DALYs by SDI quintile and GBD super-regions. The inter-category rankings show that the middle SDI quintile had the greatest DALY burden for cirrhosis and other chronic liver diseases; gallbladder and biliary diseases; inguinal, femoral, and abdominal hernia; and other digestive diseases. Compared with other SDI quintiles, the DALY burden in the high SDI quintile was ranked lowest for more than half of the digestive disease types. However, the DALY burden of inflammatory bowel disease was ranked first in the high SDI quintile. South Asia, Southeast Asia, East Asia, and Oceania had the greatest DALY burden for more digestive disease types than any other super-region. The intra-category rankings highlight that for all GBD super-regions and SDI quintiles, cirrhosis and other chronic liver diseases had the highest estimated DALY burden of all digestive disease types.

**Figure 5:**
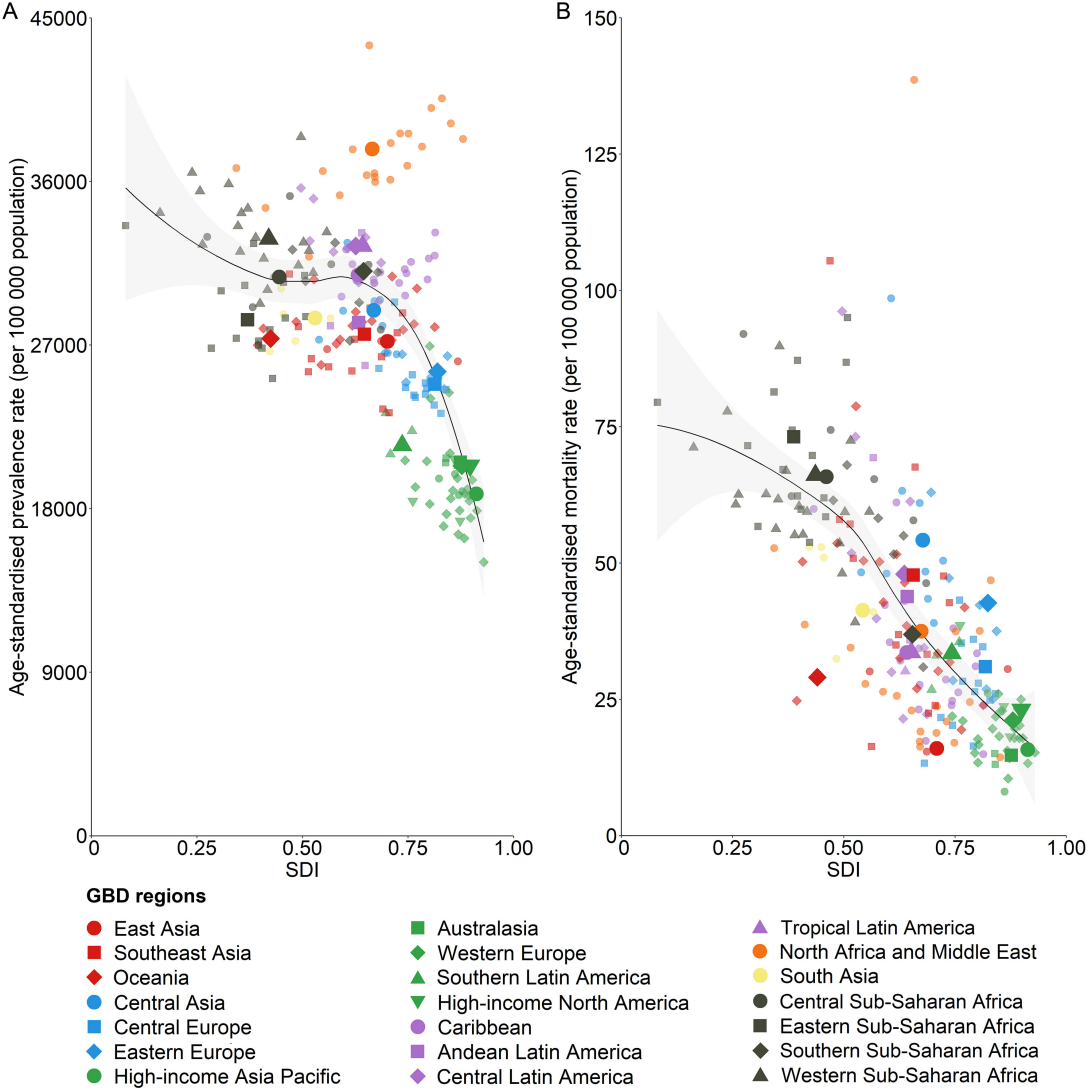
The association between SDI and digestive disease age-standardized prevalence rate **(A)** and mortality rate **(B)** for both sexes combined, 2019. Each color represents one of the seven GBD super-regions (red represents southeast Asia, east Asia, and Oceania; blue represents central Europe, eastern Europe, and central Asia; green represents high-income; purple represents Latin America and the Caribbean; orange represents North Africa and the Middle East; yellow represents south Asia; and gray represents sub-Saharan Africa). Lighter-colored point estimates without labels in the legend represent countries. The black lines represent locally weighted smoothing based on country-level data, and the gray shading represents locally weighted smoothing of country-level 95% uncertainty intervals. GBD, Global Burden of Disease, Injuries, and Risk Factors Study; SDI, Sociodemographic Index.

Focusing on the representation of digestive disease burden in terms of DALYs is not meant to devalue the importance of other disease burden metrics. As presented in the Table 1, digestive diseases accounted for 2276.27 million prevalent cases (95% UI 2151.23–2398.06) and 2.56 million deaths (2.39–2.72) in 2019, which represent 32.04% of the global non-communicable disease prevalence and 6.08% of all non-communicable disease-related deaths. The relationship between country-level age-standardized digestive disease prevalence or mortality rates and SDI is shown in Figure 5. With increasing SDI, age-standardized prevalence rates and mortality rates both decreased.

The present study depicts the contribution of four risk factors, namely smoking, alcohol use, drug use, and high body-mass index to all-age DALYs caused by digestive diseases across 21 GBD regions in 2019, as presented in Supplementary Figure 6 and Supplementary Table 6. The findings reveal that globally, smoking accounted for 1.68% of DALYs, while alcohol use and drug use contributed to 26.93 and 7.71% of DALYs, respectively, with a higher prevalence in males as compared to females. Moreover, high body-mass index was responsible for 2.30% of DALYs, with a higher prevalence observed in females. The impact of these risk factors varied across regions. In males, smoking and high body-mass index had the most significant impact in East Asia (5.71% of DALYs were attributed to smoking) and Australasia (2.70%), respectively. For females, the highest impact of smoking and high body-mass index was observed in Oceania (2.80%) and Central Latin America (9.32%), respectively. Furthermore, the impacts of alcohol and drug use were highest in Eastern Europe and High-income North America for both genders.

## Discussion

In this study, we provided the first complete and most up-to-date report detailing the current state of non-malignant digestive disease burden according to age, sex, SDI, and geographical region, by compiling the most updated statistics from GBD 2019. Notably, our analysis included assessments of certain diseases and regions that had not been previously studied. Unlike most of the previous reports focusing on disease hospitalization or mortality (16, 17), our study reported the disease burden quantified by DALYs, which can be utilized to contextualize burden in various diseases. The global age-standardized DALY rate of nonmalignant digestive disease has decreased since 1990. However, the absolute number of DALYs continued to increase, and the ranking was advanced from 15th in 1990 to 13th in 2019 among the leading causes of DALYs. The discrepancy between the increased absolute DALY number and decreased age-standardized DALY rate reflects a higher patient proportion in older age groups and global population aging (10). The highest proportion of DALYs was for cirrhosis and other chronic liver diseases, followed by upper digestive system diseases. DALYs were largely related to socioeconomic development status and mainly existed in middle-SDI countries.

We found variations related to age and sex. The 50–59 year age group had the most significant contribution to DALYs and YLLs, and the 45–54 year age group had the most significant contribution to YLDs. DALYs were generally higher in males. It was reported that hepatitis B virus and alcohol-related cirrhosis were higher in males (7), which could be driven by high-risk behaviors and alcohol consumption in males (18). Our data showed that sex also affected chronic pancreatitis, which was largely related to the reported influence of etiology and genotype on chronic pancreatitis (19–21).

Non-malignant digestive disease represented 32% of the global non-communicable disease prevalence, and the DALYs were expected to continue, leading to rising expenditures (1). The burden was affected by country SDI levels. Our data indicated that DALYs were mainly concentrated in middle-, low-middle-, and high-middle-SDI countries. In addition, high SDI countries were low in disease burden except for inflammatory bowel disease (IBD), which was due to IBD etiology and genotype, consistent with several other reports (22–24). Low-SDI countries showed a relatively low DALYs burden, which might be partly due to an underestimation caused by low epidemiological coverage, low diagnostic capacity, weak disease screening systems, and limited access to health care in resource-limited locations. Together, these data suggest the need to improve disease screening, prevention, effective intervention, and health care, especially in middle-SDI countries (17).

Our data suggested that 51.9% of digestive diseases DALYs were attributable to cirrhosis and other chronic liver diseases, which were 46.19 million DALYs and ranked 16th among the top 20 leading causes. This is consistent with several other reports (7, 25). DALYs due to cirrhosis have increased globally, specifically in low- and middle-income countries (7), which was in line with our findings. In addition, more than half of the deaths due to all non-malignant digestive diseases were attributable to liver diseases (1, 26). In the GBD super-region, the greatest absolute burden was in South Asia, Southeast Asia and East Asia, mainly due to hepatitis B virus infection (27). The greatest proportion of cirrhosis was in Central Asia and primarily due to alcoholic liver diseases (28, 29). The absolute number of DALYs of liver disorder in high-income North America was not as high as that in South Asia. However, liver disorders were the most common cause of deaths in the U.S. (1–3, 30). Taken together, these data reflected the heavy burden of liver disorders globally.

A total of 16.71% of DALYs were attributable to upper digestive system diseases, which were mainly esophageal disorders and ulcers. The age-standardized rates of the two diseases were decreased and negatively related to SDI levels (6, 31, 32). Ulcers were higher in males, and the prevalence increased with age, which was likely due to Helicobacter pylori infection and drug usage (33, 34). The incidence of pancreatitis has increased globally, and several risk factors are associated (19). Pancreatitis was mainly distributed in high-middle-SDI countries and correlating with alcohol consumption (35).

The deleterious impact of excess weight, smoking, alcohol consumption, and drug use on digestive disorders has been well-established (36–39). Our data showed that the disease burden associated with specific risk factors exhibits variation across different nations. It is noteworthy that lifestyle-related risk factors, such as smoking, sedentary behavior, and elevated body mass index (BMI), are within the purview of individual control. Moreover, public policies aimed at mitigating hazardous risk factors are imperative for minimizing the negative impact on public health. Given that the GBD database exclusively offers the rationales for four risk factors, our analytical capacity is limited to these four factors. It is imperative to analyze additional risk factors for digestive diseases after their inclusion in the GBD database.

Our study reported the most up-to-date global burden of non-malignant digestive diseases over the last 30 years and covered 204 countries and territories, some of which were not assessed before. However, there are several limitations. First, despite the considerable amount of data incorporated in the study, some estimates relied on the sparse datasets from predictive covariates or neighboring countries, especially in low-SDI countries. These data should be interpreted carefully, and high-quality epidemiological investigation should be performed in the future. Second, the digestive diseases included in GBD 2019 were those with sufficient epidemiological data at a global level required for the disease burden analysis. Other digestive diseases were limited in available resources. Efforts to build the required datasets of these diseases in the GBD study are needed. Third, the variability in study methodology also affected the precision of the estimates, which was only partially addressed in our analysis. Lack of standardization in data collection remained a major limitation. Global standards for data records using similar methodologies, data definitions, and timescales should be adopted (40). Fourth, the definition of disability was derived from a simple description, without considering the comorbidity and complexity of the disease. Estimation for YLDs considered only health loss, not welfare loss or complete disability. Further studies are needed.

## Conclusion

To summarize, our study has demonstrated an escalating burden of disability-adjusted life years (DALYs) associated with non-malignant digestive diseases on a global scale, which exhibits variability in accordance with levels of socioeconomic development and certain risk factors. Upper digestive system diseases, cirrhosis and other chronic liver diseases constituted the highest proportion of digestive disease DALY burden globally, which continued to increase and disproportionately affected low- to middle-SDI countries, underlining the need for effective disease-control strategies in these locations. It is to be hoped that the data in the report will be helpful for researchers, clinicians, policymakers, and the global health community for priority setting, resource allocation and decision making in the future.

## Data availability statement

The original contributions presented in the study are included in the article/Supplementary material, further inquiries can be directed to the corresponding author.

## Ethics statement

The institutional review board of the Third Xiangya Hospital of Central South University in Hunan Province, China, determined that the study did not need approval because it used publicly available data.

## Author contributions

Study concept and design and drafting the manuscript: DZ and RW. Acquisition of data: ZL and SL. Data analysis and interpretation and critical revision of manuscript: RW, ZL, SL, and DZ. All authors had full access to all the data in the study and had final responsibility for the decisions to submit for publication.
