# Supplementary material for: Global, regional, and national burden of 10 digestive diseases in 204 countries and territories from 1990 to 2019
Source: Front Public Health. 2023 Mar 28;11:1061453. doi: 10.3389/fpubh.2023.1061453 (PMC10088561; doi:10.3389/fpubh.2023.1061453)
Supplement: Supplementary file 1 [file Data_Sheet_1.pdf]

Supplement to: **Global, regional, and national burden of 10 digestive diseases in 204 countries and territories from 1990 to 2019**

Contents:

Supplementary Figures

Supplementary Figure 1: GBD 2019 cause hierarchy for digestive diseases.

Supplementary Figure 2: Proportion of global digestive diseases DALYs attributable to each disorder for both sexes and all ages in 2019.

Supplementary Figure 3: All-cause rankings of DALYs for digestive diseases for both sexes by all ages and five age groups in 2019.

Supplementary Figure 4: All-cause rankings of DALYs for digestive diseases for males by all ages and five age groups in 2019.

Supplementary Figure 5: All-cause rankings of DALYs for digestive diseases for females by all ages and five age groups in 2019.

Supplementary Figure 6: Percentage contribution of risk factors to all-age DALYs of digestive diseases in 2019 for males and females, globally and by regions.

Supplementary Tables

Supplementary Table 1: Digestive diseases included in GBD 2019.

Supplementary Table 2: Incident cases, years of life lost (YLLs), years lived with disability (YLDs) and their age-standardised rates due to digestive diseases in 2019 for both sexes.

Supplementary Table 3: Disability-adjusted life-years (DALYs) and age-standardised DALY rate due to digestive diseases in 2019 for both sexes by location.

Supplementary Table 4: Temporal trend in various age-standardised rates of digestive diseases globally and in 204 countries or territories from 1990 to 2019.

Supplementary Table 5: Disability-adjusted life-years (DALYs), with 95% uncertainty intervals, due to digestive diseases in 2019 for both sexes by SDI quintile and location.

Supplementary Table 6: Percentage contribution of risk factors to all-age disability-adjusted life-years (DALYs) of digestive diseases in 2019 for both sexes, globally and by regions.

Supplementary Figure 1 GBD 2019 cause hierarchy for digestive diseases

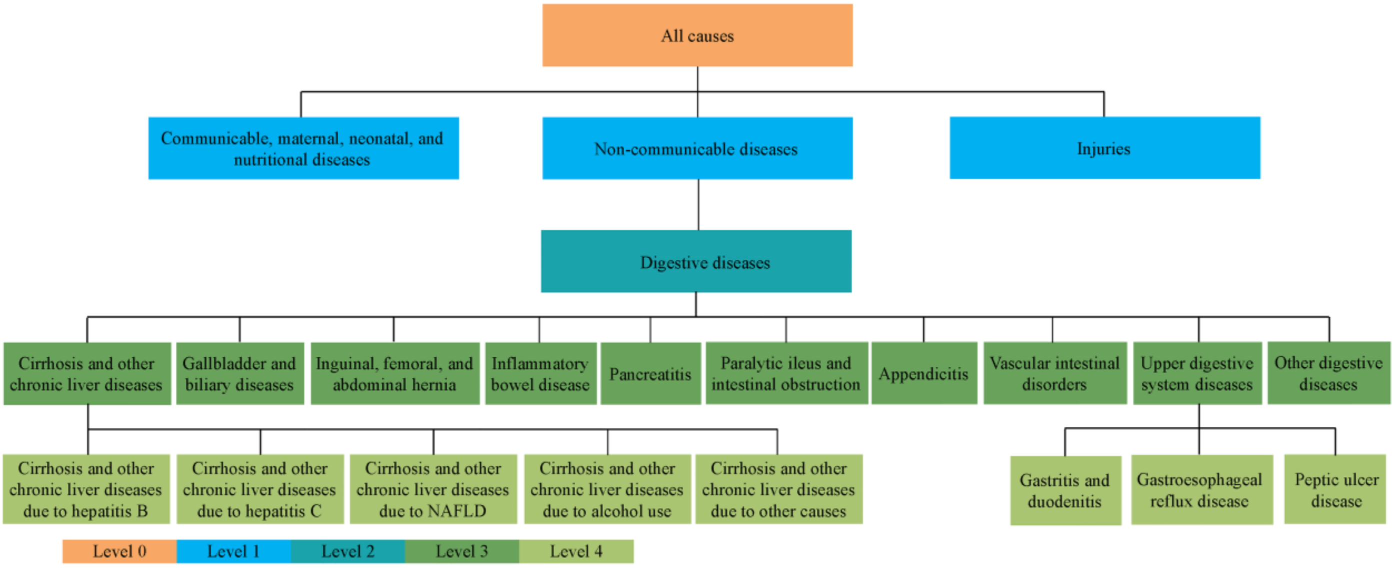

Abbreviation: GBD, Global Burden of Diseases, Injuries, and Risk Factors Study.

Supplementary Figure 2 Proportion of global digestive diseases DALYs attributable to each disorder for both sexes and all ages in 2019

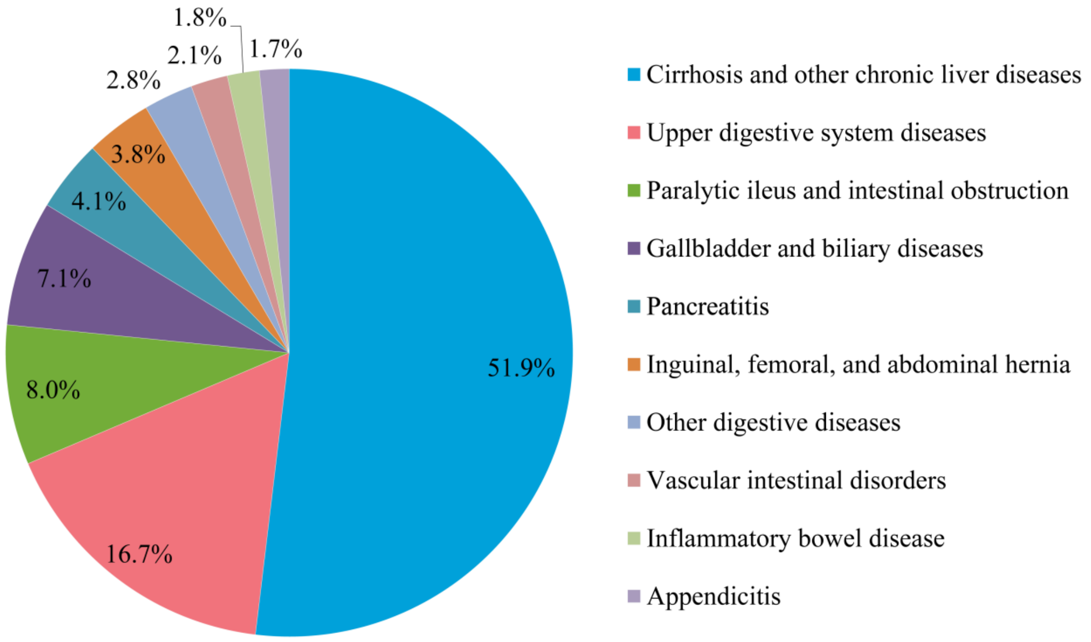

Abbreviation: DALYs, disability-adjusted life-years.

Supplementary Figure 3 All-cause rankings of DALYs for digestive diseases for both sexes by all ages and five age groups in 2019

DALYs

All ages	0-14 years	15-24 years	25-49 years	50-69 years	≥70 years
16 Cirrhosis and other chronic liver diseases	37 Paralytic ileus and intestinal obstruction	28 Cirrhosis and other chronic liver diseases	12 Cirrhosis and other chronic liver diseases	7 Cirrhosis and other chronic liver diseases	17 Cirrhosis and other chronic liver diseases
43 Upper digestive system diseases	52 Cirrhosis and other chronic liver diseases	47 Upper digestive system diseases	32 Upper digestive system diseases	34 Upper digestive system diseases	34 Upper digestive system diseases
70 Paralytic ileus and intestinal obstruction	69 Inguinal, femoral, and abdominal hernia	69 Paralytic ileus and intestinal obstruction	63 Gallbladder and biliary diseases	58 Gallbladder and biliary diseases	46 Gallbladder and biliary diseases
75 Gallbladder and biliary diseases	82 Upper digestive system diseases	85 Appendicitis	67 Pancreatitis	69 Paralytic ileus and intestinal obstruction	49 Paralytic ileus and intestinal obstruction
99 Pancreatitis	88 Other digestive diseases	92 Gallbladder and biliary diseases	76 Paralytic ileus and intestinal obstruction	79 Pancreatitis	61 Vascular intestinal disorders
103 Inguinal, femoral, and abdominal hernia	90 Appendicitis	94 Inguinal, femoral, and abdominal hernia	97 Inguinal, femoral, and abdominal hernia	85 Inguinal, femoral, and abdominal hernia	65 Other digestive diseases
115 Other digestive diseases	103 Gallbladder and biliary diseases	101 Pancreatitis	107 Inflammatory bowel disease	96 Other digestive diseases	80 Pancreatitis
127 Vascular intestinal disorders	110 Inflammatory bowel disease	117 Other digestive diseases	116 Appendicitis	102 Vascular intestinal disorders	84 Inguinal, femoral, and abdominal hernia
133 Inflammatory bowel disease	117 Pancreatitis	121 Inflammatory bowel disease	118 Other digestive diseases	111 Inflammatory bowel disease	97 Inflammatory bowel disease
135 Appendicitis	123 Vascular intestinal disorders	141 Vascular intestinal disorders	141 Vascular intestinal disorders	128 Appendicitis	124 Appendicitis

Note: This table shows DALYs rankings for each digestive disease which is ranked out of all Level 3 causes within the GBD study. Disorders are ordered from highest to lowest ranking for the overall age group (ie, all ages). Each colour represents a different digestive disease. DALYs=disability-adjusted life-years. GBD=Global Burden of Diseases, Injuries, and Risk Factors Study.

Supplementary Figure 4 All-cause rankings of DALYs for digestive diseases for males by all ages and five age groups in 2019

DALYs for males

All ages	0-14 years	15-24 years	25-49 years	50-69 years	⩾70 years
9 Cirrhosis and other chronic liver diseases	36 Paralytic ileus and intestinal obstruction	25 Cirrhosis and other chronic liver diseases	4 Cirrhosis and other chronic liver diseases	6 Cirrhosis and other chronic liver diseases	16 Cirrhosis and other chronic liver diseases
44 Upper digestive system diseases	52 Cirrhosis and other chronic liver diseases	49 Upper digestive system diseases	29 Upper digestive system diseases	34 Upper digestive system diseases	32 Upper digestive system diseases
65 Paralytic ileus and intestinal obstruction	64 Inguinal, femoral, and abdominal hernia	68 Paralytic ileus and intestinal obstruction	53 Pancreatitis	61 Paralytic ileus and intestinal obstruction	46 Paralytic ileus and intestinal obstruction
85 Pancreatitis	83 Upper digestive system diseases	86 Inguinal, femoral, and abdominal hernia	66 Paralytic ileus and intestinal obstruction	67 Gallbladder and biliary diseases	47 Gallbladder and biliary diseases
86 Inguinal, femoral, and abdominal hernia	88 Other digestive diseases	91 Appendicitis	76 Inguinal, femoral, and abdominal hernia	69 Inguinal, femoral, and abdominal hernia	63 Vascular intestinal disorders
94 Gallbladder and biliary diseases	90 Appendicitis	92 Pancreatitis	79 Gallbladder and biliary diseases	70 Pancreatitis	66 Inguinal, femoral, and abdominal hernia
112 Other digestive diseases	106 Gallbladder and biliary diseases	104 Gallbladder and biliary diseases	106 Other digestive diseases	91 Other digestive diseases	67 Other digestive diseases
124 Vascular intestinal disorders	109 Inflammatory bowel disease	112 Other digestive diseases	107 Inflammatory bowel disease	100 Vascular intestinal disorders	75 Pancreatitis
127 Inflammatory bowel disease	112 Vascular intestinal disorders	118 Inflammatory bowel disease	116 Appendicitis	110 Inflammatory bowel disease	97 Inflammatory bowel disease
132 Appendicitis	124 Pancreatitis	135 Vascular intestinal disorders	139 Vascular intestinal disorders	127 Appendicitis	117 Appendicitis

Note: This table shows DALYs rankings for each digestive disease which is ranked out of all Level 3 causes within the GBD study. Disorders are ordered from highest to lowest ranking for the overall age group (ie, all ages). Each colour represents a different digestive disease. DALYs=disability-adjusted life-years. GBD=Global Burden of Diseases, Injuries, and Risk Factors Study.

Supplementary Figure 5 All-cause rankings of DALYs for digestive diseases for females by all ages and five age groups in 2019

DALYs for females

All ages	0-14 years	15-24 years	25-49 years	50-69 years	⩾70 years
26<br>Cirrhosis and other chronic liver diseases	43<br>Paralytic ileus and intestinal obstruction	31<br>Cirrhosis and other chronic liver diseases	23<br>Cirrhosis and other chronic liver diseases	14<br>Cirrhosis and other chronic liver diseases	20<br>Cirrhosis and other chronic liver diseases
42<br>Upper digestive system diseases	54<br>Cirrhosis and other chronic liver diseases	41<br>Upper digestive system diseases	33<br>Upper digestive system diseases	35<br>Upper digestive system diseases	31<br>Upper digestive system diseases
59<br>Gallbladder and biliary diseases	79<br>Upper digestive system diseases	66<br>Paralytic ileus and intestinal obstruction	48<br>Gallbladder and biliary diseases	42<br>Gallbladder and biliary diseases	43<br>Gallbladder and biliary diseases
71<br>Paralytic ileus and intestinal obstruction	82<br>Inguinal, femoral, and abdominal hernia	73<br>Appendicitis	79<br>Paralytic ileus and intestinal obstruction	66<br>Paralytic ileus and intestinal obstruction	49<br>Paralytic ileus and intestinal obstruction
108<br>Other digestive diseases	86<br>Appendicitis	82<br>Gallbladder and biliary diseases	100<br>Pancreatitis	80<br>Pancreatitis	55<br>Vascular intestinal disorders
109<br>Pancreatitis	88<br>Other digestive diseases	108<br>Pancreatitis	104<br>Inflammatory bowel disease	90<br>Other digestive diseases	62<br>Other digestive diseases
121<br>Vascular intestinal disorders	93<br>Gallbladder and biliary diseases	112<br>Inguinal, femoral, and abdominal hernia	108<br>Appendicitis	101<br>Vascular intestinal disorders	78<br>Pancreatitis
123<br>Inguinal, femoral, and abdominal hernia	110<br>Inflammatory bowel disease	116<br>Other digestive diseases	120<br>Inguinal, femoral, and abdominal hernia	108<br>Inflammatory bowel disease	89<br>Inflammatory bowel disease
125<br>Inflammatory bowel disease	114<br>Pancreatitis	117<br>Inflammatory bowel disease	121<br>Other digestive diseases	111<br>Inguinal, femoral, and abdominal hernia	95<br>Inguinal, femoral, and abdominal hernia
126<br>Appendicitis	134<br>Vascular intestinal disorders	139<br>Vascular intestinal disorders	139<br>Vascular intestinal disorders	124<br>Appendicitis	121<br>Appendicitis

Note: This table shows DALYs rankings for each digestive disease which is ranked out of all Level 3 causes within the GBD study. Disorders are ordered from highest to lowest ranking for the overall age group (ie, all ages). Each colour represents a different digestive disease. DALYs=disability-adjusted life-years. GBD=Global Burden of Diseases, Injuries, and Risk Factors Study.

Supplementary Figure 6 Percentage contribution of risk factors to all-age DALYs of digestive diseases in 2019 for males and females, globally and by regions

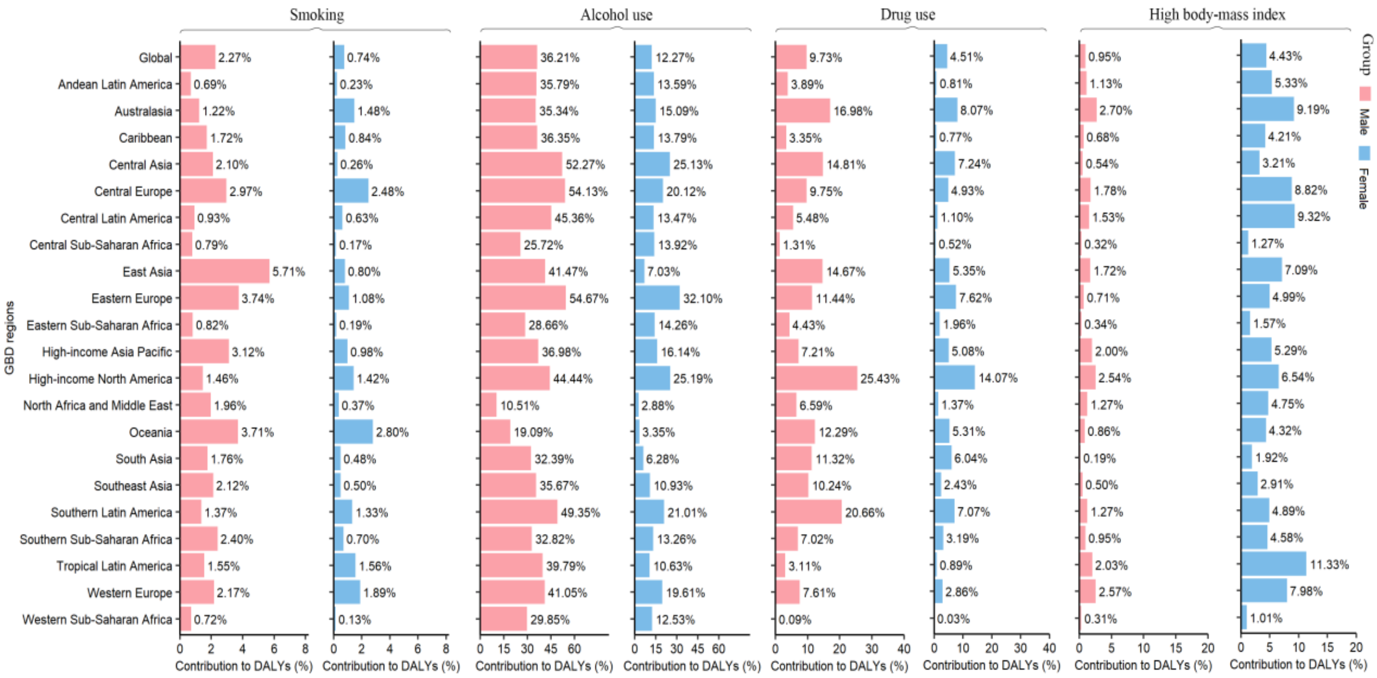

Abbreviation: DALYs, disability-adjusted life-years.

Supplementary Table 1 Digestive diseases included in GBD 2019

**Cause**	**Corresponding ICD-10 codes**
Cirrhosis and other chronic liver diseases	I85-I85.9, I98.2, K70-K71, K71.3-K72, K72.1-K75, K75.2, K75.4-K76.2, K76.4-K77.8, R16-R18.9, Z52.6, Z94.4
Upper digestive system diseases	K21-K21.9, K22.7-K22.719, K25-K30, R12
Gallbladder and biliary diseases	K80-K80.81, K81-K83.9, K87-K87.1
Inguinal, femoral, and abdominal hernia	K40-K42.9, K44-K46.9
Inflammatory bowel disease	K50-K51.319, K51.5-K52, K52.8-K52.9
Pancreatitis	K85-K86.9
Appendicitis	K35-K37.9
Paralytic ileus and intestinal obstruction	K56-K56.9
Vascular intestinal disorders	K55-K55.9
Other digestive diseases	I84-I84.9, K20-K20.9, K22-K22.6, K22.8-K23.8, K31-K31.9, K38-K38.9, K52.2-K52.3, K57-K62, K62.4-K62.6, K62.8-K63.4, K63.8-K67, K67.8-K68.1, K68.12-K68.9, K71.0-K71.2, K72.0-K72.01, K90-K90.9, K92-K92.9, K93.8

Abbreviations: GBD, Global Burden of Diseases, Injuries, and Risk Factors Study; ICD-10, 10th revision of the International Classification of Diseases.

Supplementary Table 2 Incident cases, years of life lost (YLLs), years lived with disability (YLDs) and their age-standardised rates due to digestive diseases in 2019 for both sexes

Characteristics	Incident cases, in millions (95% UI)	Age-standardised incidence rate per 100,000 population (95% UI)	YLLs, in millions (95% UI)	Age-standardised YLL rate per 100,000 population (95% UI)	YLDs, in millions (95% UI)	Age-standardised YLD rate per 100,000 population (95% UI)
**Global**	443.53 (405.58-484.42)	5454.63 (4988.69-5942.26)	71.97 (67.01-77.12)	887.74 (825.82-952.74)	17.02 (11.91-23.88)	209.25 (146.37-292.88)
**SDI quintile**						
High	68.28 (62.79-74.60)	5237.73 (4805.17-5740.57)	7.80 (7.39-8.09)	481.08 (460.92-497.08)	2.72 (1.94-3.75)	206.43 (146.91-286.53)
High-middle	91.02 (83.78-99.72)	5207.59 (4786.46-5672.83)	11.79 (11.08-12.53)	625.52 (587.62-664.66)	3.66 (2.58-5.08)	206.11 (144.37-286.60)
Middle	131.48 (120.21-143.83)	5079.07 (4650.21-5522.56)	20.06 (18.48-21.89)	794.36 (729.68-868.12)	5.09 (3.53-7.13)	196.64 (136.75-275.03)
Low-middle	103.46 (93.82-113.47)	6198.92 (5661.78-6770.73)	20.33 (18.49-22.35)	1306.49 (1191.92-1431.91)	3.80 (2.63-5.36)	231.29 (160.54-325.99)
Low	49.04 (43.99-54.36)	5905.23 (5348.25-6503.46)	11.94 (10.28-13.75)	1602.31 (1409.88-1812.10)	1.75 (1.18-2.52)	211.58 (144.31-303.32)
**Disorders**						
Cirrhosis and other chronic liver diseases	2.05 (1.66-2.48)	25.35 (20.78-30.44)	45.49 (42.33-48.79)	551.86 (513.55-592.55)	0.69 (0.48-0.97)	8.57 (5.93-12.03)
Upper digestive system diseases	343.97 (305.20-383.74)	4216.93 (3734.36-4713.11)	6.47 (6.02-7.08)	79.98 (74.35-87.33)	8.40 (4.93-13.77)	102.83 (60.37-168.66)
Gallbladder and biliary diseases	52.00 (44.20-61.21)	634.32 (540.21-742.93)	2.29 (1.97-2.52)	28.92 (24.89-31.76)	4.06 (2.60-5.95)	49.33 (31.51-71.96)
Inguinal, femoral, and abdominal hernia	13.02 (10.68-15.49)	162.96 (134.12-192.86)	1.16 (0.99-1.32)	15.12 (12.89-17.25)	2.20 (1.47-3.07)	27.62 (18.49-38.67)
Inflammatory bowel disease	0.40 (0.36-0.46)	4.97 (4.43-5.59)	0.90 (0.78-1.00)	11.35 (9.89-12.70)	0.73 (0.49-0.99)	8.80 (5.90-12.05)
Pancreatitis	2.81 (2.41-3.29)		3.45 (3.12-3.82)	42.04 (38.06-46.59)	0.19 (0.11-0.30)	2.36 (1.39-3.70)
Appendicitis	17.70 (14.10-22.32)	229.86 (180.88-291.00)	1.29 (1.03-1.47)	16.62 (13.29-18.96)	0.21 (0.14-0.30)	2.74 (1.76-3.93)
Paralytic ileus and intestinal obstruction	10.10 (9.75-10.44)	127.24 (122.92-131.33)	6.91 (5.50-8.08)	91.18 (71.89-107.28)	0.17 (0.11-0.23)	2.28 (1.55-3.10)
Vascular intestinal disorders	1.46 (1.26-1.65)	18.23 (15.84-20.67)	1.82 (1.66-1.98)	22.84 (20.83-24.96)	0.05 (0.03-0.07)	0.65 (0.44-0.90)

Other digestive diseases	NA	NA	2.18 (1.83-2.46)	28.92 (24.89-31.76)	0.32 (0.22-0.43)	4.07 (2.86-5.47)

Abbreviations: NA, not available; SDI, Socio-demographic Index; UI, uncertainty interval.

Supplementary Table 3 Disability-adjusted life-years (DALYs) and age-standardised DALY rate due to digestive diseases in 2019 for both sexes by location

Location	Age-standardised DALY rate per 100,000 population	95% uncertainty intervals (age-standardised DALY rate)	DALYs, in thousands	95% uncertainty intervals (DALYs)
**Global**	1096.99	(1002.19-1202.93)	88991.77	(81413.80 -97583.80)
**Central Europe, eastern Europe, and central Asia**	1522.18	(1399.12-1670.12)	8172.32	(7510.57 -8934.15)
**Central Asia**	1827.87	(1659.55-2024.27)	1615.95	(1458.62 -1799.79)
Armenia	1191.47	(1024.80-1365.71)	45.28	(38.85 -52.07)
Azerbaijan	1355.69	(1152.56-1600.73)	136.73	(116.12 -162.97)
Georgia	1402.03	(1197.47-1607.87)	68.10	(58.13 -78.01)
Kazakhstan	1717.00	(1477.71-2076.18)	326.98	(279.86 -397.74)
Kyrgyzstan	1756.86	(1556.82-1977.68)	101.28	(89.64 -114.20)
Mongolia	2731.15	(2202.64-3420.59)	79.67	(63.39 -100.39)
Tajikistan	1603.65	(1337.62-1918.74)	117.06	(97.64 -140.34)
Turkmenistan	2368.94	(1961.34-2903.24)	116.71	(96.55 -143.15)
Uzbekistan	2133.56	(1834.73-2488.83)	624.15	(533.26 -731.50)
**Central Europe**	1100.47	(966.13-1241.20)	1895.69	(1669.10 -2127.18)
Albania	567.46	(462.43-689.47)	19.44	(15.67 -23.94)
Bosnia and Herzegovina	762.91	(623.20-923.66)	38.77	(31.62 -47.05)
Bulgaria	1281.70	(1045.07-1554.49)	138.91	(112.99 -168.15)
Croatia	897.56	(731.13-1082.51)	62.55	(51.21 -75.14)
Czechia	895.75	(747.99-1061.79)	147.77	(123.77 -174.82)
Hungary	1269.02	(1062.81-1489.21)	194.78	(164.07 -228.25)
Montenegro	658.87	(553.16-799.41)	5.59	(4.69 -6.75)
North Macedonia	728.05	(599.06-880.14)	21.14	(17.34 -25.80)
Poland	1032.81	(874.30-1210.55)	579.38	(489.85 -677.59)
Romania	1500.95	(1268.79-1781.88)	443.34	(374.77 -525.95)
Serbia	864.99	(713.10-1043.16)	116.17	(96.02 -139.75)
Slovakia	1241.80	(995.88-1555.08)	97.94	(78.67 -122.76)
Slovenia	864.30	(697.49-1078.43)	29.92	(24.43 -37.37)
**Eastern Europe**	1651.18	(1480.22-1844.63)	4660.68	(4188.63 -5200.98)
Belarus	1156.63	(939.59-1448.37)	152.56	(123.86 -190.50)
Estonia	987.15	(805.09-1202.64)	18.81	(15.39 -23.00)
Latvia	1069.09	(903.25-1256.41)	30.30	(25.70 -35.60)
Lithuania	1447.22	(1215.15-1729.25)	59.31	(49.81 -70.77)
Republic of Moldova	2181.73	(1920.76-2457.64)	114.77	(100.80 -129.10)
Russian Federation	1579.40	(1382.58-1801.80)	3108.30	(2728.17 -3549.47)
Ukraine	2007.17	(1713.04-2356.30)	1176.63	(1004.33 -1377.25)
**High income**	686.34	(624.54-764.67)	11519.07	(10555.38 -12623.70)
**Australasia**	471.87	(414.16-544.76)	196.22	(174.53 -222.06)
Australia	474.11	(417.35-545.27)	166.49	(148.21 -188.12)

New Zealand	461.67	(392.46-551.72)	29.73	(25.80 -34.85)
**High-income Asia Pacific**	566.92	(491.78-650.77)	1843.64	(1635.46 -2063.38)
Brunei Darussalam	747.05	(644.70-856.34)	2.76	(2.34 -3.20)
Japan	547.23	(470.73-634.77)	1302.46	(1143.27 -1461.36)
Republic of Korea	645.95	(565.45-738.19)	512.62	(453.08 -582.02)
Singapore	355.90	(297.75-431.79)	25.80	(21.66 -31.18)
**High-income North America**	772.53	(711.01-851.74)	3999.01	(3715.47 -4356.95)
Canada	584.64	(520.12-659.06)	330.57	(298.46 -367.33)
Greenland	1127.49	(929.32-1369.56)	0.78	(0.64 -0.95)
United States of America	794.44	(732.92-873.74)	3667.59	(3408.83 -3990.29)
**Southern Latin America**	965.63	(888.72-1064.68)	757.57	(699.87 -831.96)
Argentina	953.24	(873.46-1055.94)	486.29	(448.17 -537.15)
Chile	1032.03	(949.41-1133.06)	235.91	(217.54 -256.99)
Uruguay	748.37	(675.02-841.14)	35.34	(32.19 -39.20)
**Western Europe**	652.28	(589.37-731.34)	4722.63	(4315.93 -5176.79)
Andorra	542.21	(431.22-671.75)	0.71	(0.56 -0.88)
Austria	673.55	(595.49-767.96)	94.48	(84.55 -105.65)
Belgium	640.59	(579.54-713.52)	120.78	(110.47 -131.91)
Cyprus	483.39	(422.64-555.12)	8.44	(7.36 -9.69)
Denmark	685.50	(615.56-767.94)	63.68	(57.83 -70.01)
Finland	788.04	(716.95-873.06)	68.86	(63.01 -75.33)
France	565.42	(511.55-628.71)	616.28	(562.07 -672.07)
Germany	742.25	(676.51-816.27)	1105.17	(1018.08 -1198.64)
Greece	469.63	(412.15-547.83)	87.73	(78.34 -99.53)
Iceland	354.88	(305.37-411.01)	1.68	(1.46 -1.92)
Ireland	465.49	(413.42-533.31)	31.00	(27.73 -35.02)
Israel	446.77	(393.29-513.09)	48.18	(42.86 -54.81)
Italy	643.64	(553.39-751.80)	700.55	(618.49 -791.37)
Luxembourg	610.19	(530.82-700.44)	5.48	(4.82 -6.26)
Malta	429.32	(371.34-494.70)	3.08	(2.71 -3.50)
Netherlands	437.86	(394.15-490.99)	127.79	(116.26 -140.93)
Norway	509.64	(431.21-606.07)	40.34	(34.75 -46.83)
Portugal	624.49	(570.54-690.10)	117.54	(108.33 -127.99)
Monaco	559.06	(452.05-669.93)	0.39	(0.32 -0.46)
San Marino	550.24	(401.99-752.90)	0.30	(0.22 -0.41)
Spain	551.46	(499.74-614.23)	453.57	(414.41 -494.92)
Sweden	465.66	(409.76-534.64)	76.63	(68.42 -85.86)
Switzerland	426.62	(381.35-476.14)	62.98	(56.48 -69.30)
United Kingdom	889.15	(793.83-1008.37)	882.86	(796.49 -984.88)
**Latin America and Caribbean**	1362.86	(1220.83-1515.59)	8175.63	(7319.11 -9104.33)
**Andean Latin America**	1353.17	(1117.75-1636.97)	802.27	(662.81 -972.61)
Ecuador	1361.68	(1107.89-1682.08)	217.09	(176.22 -269.04)
Peru	1189.63	(911.62-1568.40)	394.88	(303.39 -519.48)

Bolivia (Plurinational State of)	1915.20	(1506.15-2368.25)	190.29	(149.54 -234.75)
**Caribbean**	1246.12	(1002.11-1490.81)	618.87	(502.09 -736.24)
Antigua and Barbuda	841.57	(714.65-986.03)	0.85	(0.72 -0.99)
Barbados	828.53	(690.39-992.11)	3.42	(2.86 -4.10)
Belize	1251.08	(1092.98-1425.54)	4.27	(3.72 -4.89)
Bermuda	599.11	(490.77-723.32)	0.60	(0.49 -0.72)
Bahamas	1117.75	(928.13-1352.61)	4.61	(3.81 -5.62)
Cuba	834.80	(691.76-1001.60)	137.12	(113.53 -164.17)
Dominica	989.43	(813.93-1201.68)	0.80	(0.66 -0.97)
Dominican Republic	1400.15	(1067.83-1786.31)	141.91	(107.62 -181.64)
Grenada	1147.42	(1017.84-1292.25)	1.30	(1.15 -1.46)
Guyana	2080.92	(1664.96-2564.57)	14.98	(11.94 -18.52)
Haiti	1999.90	(1263.16-2721.39)	191.27	(117.90 -267.33)
Jamaica	695.04	(561.93-848.24)	20.47	(16.53 -25.03)
Puerto Rico	860.42	(685.90-1064.22)	45.65	(36.57 -56.27)
Saint Kitts and Nevis	1194.24	(948.51-1446.63)	0.83	(0.65 -1.00)
Saint Lucia	965.12	(817.59-1130.43)	2.00	(1.69 -2.34)
Saint Vincent and the Grenadines	1124.67	(965.98-1299.01)	1.46	(1.26 -1.68)
Suriname	1408.17	(1177.38-1667.41)	8.50	(7.09 -10.06)
Trinidad and Tobago	956.63	(755.92-1201.31)	16.21	(12.77 -20.43)
United States Virgin Islands	1089.45	(904.12-1286.56)	1.67	(1.39 -1.96)
**Central Latin America**	1571.99	(1386.97-1788.67)	3874.04	(3413.09 -4405.84)
Venezuela (Bolivarian Republic of)	1055.97	(838.23-1311.26)	314.02	(248.22 -390.68)
Colombia	787.50	(629.65-966.27)	404.01	(321.81 -495.48)
Costa Rica	1100.28	(893.09-1343.56)	56.58	(45.95 -69.20)
El Salvador	1364.41	(1087.96-1702.42)	81.70	(65.41 -101.85)
Guatemala	2441.99	(1971.58-3008.48)	338.83	(273.88 -416.21)
Honduras	2725.29	(2128.51-3422.48)	198.61	(157.01 -245.95)
Mexico	1910.20	(1655.10-2189.24)	2364.01	(2040.23 -2714.24)
Nicaragua	1569.83	(1314.33-1860.05)	81.12	(67.92 -96.54)
Panama	844.73	(680.16-1029.17)	35.16	(28.32 -42.93)
**Tropical Latin America**	1177.44	(1075.26-1307.35)	2880.45	(2627.83 -3203.65)
Brazil	1180.77	(1078.77-1312.11)	2815.96	(2568.22 -3133.70)
Paraguay	1051.01	(838.66-1296.54)	64.50	(51.25 -79.37)
**North Africa and Middle East**	995.00	(796.21-1179.67)	4841.61	(3845.35 -5801.70)
Afghanistan	1474.35	(1062.19-1942.45)	319.38	(219.56 -434.25)
Algeria	636.41	(515.11-780.66)	233.28	(187.13 -289.65)
Bahrain	786.84	(631.24-982.38)	8.66	(6.77 -10.99)
Egypt	2858.13	(1898.97-3720.61)	1873.91	(1235.11 -2464.08)
Iraq	570.39	(460.58-697.31)	177.24	(142.32 -219.76)

Iran (Islamic Republic of)	549.52	(473.15-649.07)	438.71	(374.27 -522.11)
Jordan	585.78	(480.78-697.96)	47.55	(38.61 -58.17)
Kuwait	461.27	(384.74-558.24)	17.32	(14.02 -21.73)
Lebanon	568.84	(411.23-776.32)	29.82	(21.47 -40.73)
Libya	684.68	(511.67-892.08)	40.60	(29.69 -52.83)
Morocco	752.30	(610.13-892.58)	244.82	(196.13 -295.53)
Palestine	694.87	(587.67-811.22)	20.95	(17.62 -25.03)
Oman	661.09	(525.19-837.92)	18.29	(13.52 -24.58)
Qatar	906.94	(695.60-1199.31)	12.48	(9.27 -16.52)
Saudi Arabia	861.37	(695.06-1037.85)	198.77	(155.36 -247.52)
Sudan	975.59	(695.80-1397.24)	262.40	(190.84 -364.13)
Syrian Arab Republic	718.45	(543.20-909.44)	92.84	(69.99 -118.38)
Tunisia	566.49	(427.66-766.12)	69.78	(52.44 -94.57)
Turkey	550.16	(448.62-674.97)	481.14	(389.84 -594.23)
United Arab Emirates	606.87	(461.44-787.68)	44.36	(32.84 -58.86)
Yemen	1073.17	(809.03-1419.55)	204.37	(154.78 -262.42)
**South Asia**	1422.01	(1259.87-1611.05)	23225.08	(20533.08 -26276.67)
Bangladesh	1146.02	(965.49-1394.54)	1674.98	(1404.06 -2033.38)
Bhutan	1550.20	(1100.85-2762.65)	10.30	(7.22 -18.29)
India	1421.21	(1237.02-1628.55)	18467.79	(16032.08 -21139.51)
Nepal	1585.15	(1205.17-2099.86)	403.13	(307.73 -532.95)
Pakistan	1677.09	(1300.74-2220.26)	2668.88	(2082.21 -3491.25)
**Southeast Asia, east Asia, and Oceania**	770.32	(691.79-848.87)	19779.57	(17738.64 -21855.47)
**East Asia**	551.10	(476.11-627.28)	10616.91	(9139.05 -12123.52)
China	538.73	(463.28-615.65)	10017.88	(8549.91 -11496.36)
Democratic People's Republic of Korea	917.56	(643.72-1169.12)	289.28	(197.50 -370.33)
Taiwan (Province of China)	878.52	(710.34-1100.30)	309.75	(248.94 -388.33)
**Oceania**	972.14	(805.16-1172.85)	99.83	(81.75 -121.26)
American Samoa	909.95	(750.32-1090.47)	0.46	(0.38 -0.56)
Cook Islands	603.82	(495.94-737.13)	0.13	(0.11 -0.16)
Micronesia (Federated States of)	1491.13	(905.88-2216.33)	1.27	(0.74 -1.94)
Fiji	810.74	(641.32-1007.98)	6.72	(5.30 -8.44)
Guam	804.05	(677.31-946.87)	1.47	(1.23 -1.73)
Kiribati	2278.18	(1475.57-3222.87)	2.03	(1.22 -2.96)
Marshall Islands	1512.45	(982.12-2169.32)	0.69	(0.44 -1.01)
Northern Mariana Islands	1182.76	(988.41-1412.46)	0.63	(0.52 -0.74)
Papua New Guinea	874.75	(690.36-1108.02)	66.28	(51.92 -85.15)
Nauru	1537.91	(992.25-2140.98)	0.11	(0.07 -0.16)
Niue	989.69	(755.35-1280.77)	0.02	(0.02 -0.03)
Palau	988.32	(658.55-1378.48)	0.22	(0.14 -0.31)
Samoa	1120.52	(852.88-1431.56)	1.87	(1.41 -2.41)

Solomon Islands	1765.17	(1437.26-2116.72)	8.47	(6.84 -10.26)
Tokelau	948.60	(729.78-1218.01)	0.01	(0.01 -0.02)
Tonga	1297.30	(1028.55-1603.89)	1.11	(0.88 -1.37)
Tuvalu	1278.80	(893.12-1770.85)	0.14	(0.10 -0.19)
Vanuatu	1615.20	(1077.76-2204.48)	3.48	(2.34 -4.78)
**Southeast Asia**	1376.47	(1235.86-1523.75)	9062.84	(8106.34 -10089.77)
Cambodia	2937.01	(2391.93-3500.65)	407.79	(331.49 -490.74)
Indonesia	1737.19	(1510.33-1967.33)	4036.40	(3468.61 -4646.49)
Lao Peoples's Democratic Republic	1606.43	(1198.78-2108.10)	86.47	(64.38 -113.34)
Malaysia	1031.19	(832.32-1258.64)	287.90	(231.64 -355.04)
Maldives	482.56	(405.69-563.65)	2.02	(1.70 -2.40)
Mauritius	829.63	(692.10-989.37)	13.53	(11.24 -16.28)
Myanmar	1881.87	(1508.08-2329.64)	1026.87	(815.06 -1273.29)
Philippines	1096.60	(923.52-1301.89)	1013.15	(854.16 -1205.10)
Sri Lanka	721.87	(564.19-917.39)	179.19	(139.41 -228.94)
Seychelles	1386.66	(1192.02-1580.21)	1.63	(1.39 -1.87)
Viet Nam	1003.74	(806.02-1228.18)	1012.76	(806.66 -1259.00)
Thailand	1019.92	(802.56-1292.65)	969.44	(754.40 -1233.88)
Timor-Leste	1513.67	(983.72-2185.96)	13.83	(8.62 -19.68)
**Sub-Saharan Africa**	1925.55	(1639.92-2252.90)	13278.48	(11044.91 -15915.54)
**Central sub-Saharan Africa**	2010.42	(1577.63-2506.31)	1626.12	(1259.51 -2080.99)
Angola	2176.16	(1742.73-2698.78)	380.61	(285.07 -490.62)
Central African Republic	2814.31	(1994.97-3886.18)	92.65	(63.13 -131.06)
Congo	1860.70	(1361.35-2368.50)	66.55	(47.97 -87.94)
Democratic Republic of the Congo	1938.72	(1457.47-2480.81)	1054.48	(799.96 -1409.11)
Equatorial Guinea	1319.21	(947.57-1850.15)	10.08	(6.91 -14.63)
Gabon	1664.11	(1277.41-2085.89)	21.75	(16.45 -28.05)
**Eastern sub-Saharan Africa**	2065.98	(1804.74-2398.21)	4941.05	(4214.88 -5827.01)
Burundi	2112.22	(1583.41-2960.27)	145.41	(109.17 -203.69)
Comoros	1762.70	(1343.19-2237.19)	9.95	(7.39 -12.75)
Djibouti	1690.51	(1154.78-2551.51)	14.26	(9.58 -21.58)
Eritrea	2519.10	(1942.26-3276.83)	101.33	(75.63 -134.43)
Ethiopia	2184.92	(1808.49-2653.33)	1298.79	(1059.99 -1605.21)
Kenya	2507.50	(1841.17-3486.78)	735.86	(540.67 -1019.17)
Madagascar	1830.57	(1406.64-2375.38)	298.14	(229.78 -388.30)
Malawi	2166.76	(1784.78-2624.75)	230.80	(187.19 -282.34)
Mozambique	1740.44	(1386.69-2172.28)	317.23	(243.40 -403.98)
Rwanda	2007.87	(1607.10-2514.05)	166.51	(128.16 -215.49)
Somalia	2371.31	(1644.47-3363.68)	261.59	(178.30 -372.27)
South Sudan	1906.44	(1355.53-2874.39)	107.63	(77.36 -162.59)
United Republic of Tanzania	1626.46	(1303.97-1986.60)	603.32	(487.01 -750.72)
Uganda	1722.50	(1412.76-2075.22)	378.62	(300.87 -470.98)

Zambia	2539.15	(1988.30-3251.77)	267.67	(201.37 -354.23)
**Southern sub-Saharan Africa**	1167.53	(1047.65-1298.34)	787.27	(700.00 -882.83)
Botswana	1700.78	(1265.07-2220.33)	31.65	(23.12 -42.28)
Lesotho	2074.53	(1516.38-2733.21)	32.84	(23.46 -43.46)
Namibia	1525.34	(1154.58-1982.45)	26.67	(19.65 -35.21)
South Africa	976.49	(871.18-1092.19)	501.16	(446.18 -563.18)
Eswatini	1786.09	(1319.66-2393.63)	14.13	(10.28 -19.01)
Zimbabwe	1853.60	(1253.64-2427.25)	180.83	(122.70 -239.03)
**Western sub-Saharan Africa**	1982.79	(1581.80-2480.49)	5924.04	(4639.64 -7472.50)
Benin	1885.96	(1458.38-2461.63)	150.26	(111.72 -199.95)
Burkina Faso	1858.22	(1398.69-2379.60)	291.26	(216.11 -375.46)
Cameroon	1658.57	(1128.97-2255.69)	306.14	(204.04 -418.17)
Chad	2371.08	(1894.29-2929.11)	225.54	(178.65 -279.34)
Ghana	1810.88	(1454.23-2214.33)	408.61	(322.44 -500.96)
Guinea	1962.07	(1448.99-2557.79)	163.20	(116.76 -218.24)
Guinea-Bissau	2717.44	(2064.73-3445.21)	30.84	(23.28 -39.53)
Liberia	1975.93	(1400.69-2735.53)	63.14	(42.58 -92.92)
Mali	1969.62	(1487.49-2584.68)	278.40	(197.45 -385.62)
Mauritania	1409.69	(1010.92-1930.70)	37.79	(25.84 -54.03)
Niger	2177.59	(1646.13-2881.07)	299.12	(217.03 -410.24)
Nigeria	2116.08	(1392.64-3131.13)	2977.41	(1939.14 -4448.35)
Cape Verde	1189.74	(1013.67-1407.04)	5.86	(4.97 -6.98)
Côte d'Ivoire	1702.13	(1247.75-2214.43)	290.05	(209.33 -380.84)
The Gambia	1785.23	(1354.32-2257.25)	24.36	(18.77 -30.48)
Sao Tome and Principe	1743.42	(1392.57-2423.77)	2.48	(1.97 -3.48)
Senegal	1676.14	(1219.12-2225.91)	170.62	(122.90 -230.96)
Sierra Leone	1804.67	(1324.49-2382.94)	102.55	(72.48 -140.47)
Togo	1814.70	(1394.98-2285.04)	96.31	(72.34 -121.86)

Note: North Africa & Middle East and South Asia are both super-regions and regions.

Supplementary Table 4 Temporal trend in various age-standardised rates of digestive diseases globally and in 204 countries or territories from 1990 to 2019

Location	EAPC of age-standardised prevalence rate (95% CI)	EAPC of age-standardised incidence rate (95% CI)	EAPC of age-standardised mortality rate (95% CI)	EAPC of age-standardised YLL rate (95% CI)	EAPC of age-standardised YLD rate (95% CI)	EAPC of age-standardised DALY rate (95% CI)
**Global**	-0.02 (-0.07 to 0.02)	0.09 (0.05 to 0.12)	-1.38 (-1.44 to -1.31)	-1.51 (-1.57 to -1.44)	-0.38 (-0.44 to -0.33)	-1.32 (-1.36 to -1.27)
**Central Europe, eastern Europe, and central Asia**	0.02 (0.00 to 0.04)	-0.03 (-0.07 to 0.01)	0.72 (0.37 to 1.07)	0.91 (0.45 to 1.36)	-0.24 (-0.27 to -0.21)	0.70 (0.33 to 1.08)
**Central Asia**	-0.04 (-0.06 to -0.02)	0.07 (0.06 to 0.09)	0.79 (0.43 to 1.16)	0.57 (0.16 to 0.97)	0.01 (0.00 to 0.03)	0.50 (0.14 to 0.85)
Armenia	0.13 (0.12 to 0.13)	0.18 (0.16 to 0.20)	1.86 (1.37 to 2.36)	0.77 (0.32 to 1.21)	0.19 (0.14 to 0.25)	0.65 (0.29 to 1.02)
Azerbaijan	0.00 (-0.02 to 0.02)	0.07 (0.05 to 0.09)	-0.23 (-0.64 to 0.19)	-0.98 (-1.33 to -0.63)	-0.09 (-0.12 to -0.06)	-0.86 (-1.16 to -0.56)
Georgia	0.05 (0.03 to 0.08)	0.16 (0.13 to 0.19)	0.56 (0.31 to 0.82)	0.61 (0.37 to 0.85)	0.52 (0.42 to 0.63)	0.60 (0.38 to 0.81)
Kazakhstan	-0.12 (-0.14 to -0.10)	0.05 (0.04 to 0.06)	1.54 (1.04 to 2.04)	1.54 (0.86 to 2.22)	-0.11 (-0.17 to -0.05)	1.31 (0.71 to 1.91)
Kyrgyzstan	-0.10 (-0.11 to -0.08)	0.06 (0.04 to 0.08)	0.25 (-0.32 to 0.82)	0.27 (-0.33 to 0.87)	0.00 (-0.03 to 0.03)	0.24 (-0.29 to 0.77)
Mongolia	-0.30 (-0.34 to -0.26)	-0.09 (-0.12 to -0.07)	-0.59 (-0.81 to -0.37)	-1.42 (-1.57 to -1.26)	-0.80 (-0.87 to -0.73)	-1.37 (-1.51 to -1.22)
Tajikistan	-0.13 (-0.14 to -0.11)	0.06 (0.05 to 0.08)	0.47 (0.23 to 0.71)	-0.35 (-0.51 to -0.19)	0.05 (0.01 to 0.09)	-0.30 (-0.44 to -0.17)
Turkmenistan	-0.02 (-0.04 to 0.00)	0.09 (0.07 to 0.10)	0.66 (0.41 to 0.91)	1.11 (0.86 to 1.36)	0.09 (0.06 to 0.12)	1.01 (0.79 to 1.23)
Uzbekistan	-0.09 (-0.11 to -0.07)	0.10 (0.09 to 0.12)	0.82 (0.20 to 1.45)	0.54 (-0.07 to 1.15)	0.07 (0.05 to 0.09)	0.48 (-0.07 to 1.03)
**Central Europe**	-0.06 (-0.07 to -0.05)	0.02 (-0.01 to 0.05)	-1.22 (-1.35 to -1.08)	-1.47 (-1.64 to -1.30)	-0.47 (-0.50 to -0.44)	-1.25 (-1.38 to -1.12)
Albania	0.06 (0.03 to 0.08)	0.16 (0.14 to 0.18)	-2.77 (-3.17 to -2.37)	-3.44 (-3.97 to -2.90)	-0.18 (-0.23 to -0.13)	-2.46 (-2.88 to -2.05)
Bosnia and Herzegovina	0.17 (0.16 to 0.19)	0.09 (0.08 to 0.10)	-1.88 (-2.12 to -1.63)	-2.30 (-2.58 to -2.02)	-0.40 (-0.46 to -0.34)	-1.76 (-1.99 to -1.53)
Bulgaria	-0.28 (-0.30 to -0.25)	0.00 (-0.02 to 0.02)	0.08 (-0.10 to 0.26)	0.09 (-0.11 to 0.29)	-0.37 (-0.41 to -0.32)	-0.01 (-0.17 to 0.15)
Croatia	0.00 (-0.02 to 0.02)	0.08 (0.05 to 0.11)	-2.01 (-2.18 to -1.83)	-2.64 (-2.84 to -2.44)	-0.08 (-0.14 to -0.01)	-2.03 (-2.18 to -1.88)
Czechia	0.01 (0.00 to 0.03)	0.12 (0.10 to 0.14)	-1.08 (-1.23 to -0.92)	-1.28 (-1.43 to -1.13)	0.01 (-0.04 to 0.07)	-0.95 (-1.06 to -0.83)
Hungary	-0.07 (-0.08 to	0.01 (0.00 to	-3.08 (-3.40 to	-3.88 (-4.28 to	-0.12 (-0.15 to	-3.26 (-3.60 to

	-0.05)	0.02)	-2.77)	-3.48)	-0.10)	-2.92)
Montenegro	0.07 (0.05 to 0.08)	0.09 (0.07 to 0.11)	-0.08 (-0.26 to 0.10)	-0.45 (-0.66 to -0.24)	0.01 (-0.01 to 0.03)	-0.29 (-0.42 to -0.15)
North Macedonia	0.04 (0.04 to 0.05)	0.09 (0.08 to 0.10)	-0.59 (-0.70 to -0.47)	-1.08 (-1.17 to -0.98)	-0.19 (-0.22 to -0.15)	-0.80 (-0.87 to -0.74)
Poland	-0.14 (-0.17 to -0.11)	-0.19 (-0.26 to -0.12)	-0.82 (-0.97 to -0.67)	-0.45 (-0.65 to -0.24)	-1.13 (-1.20 to -1.05)	-0.63 (-0.78 to -0.48)
Romania	0.01 (0.00 to 0.02)	0.12 (0.10 to 0.14)	-0.74 (-1.03 to -0.46)	-1.08 (-1.41 to -0.75)	-0.18 (-0.22 to -0.14)	-0.94 (-1.21 to -0.66)
Serbia	0.06 (0.06 to 0.07)	0.26 (0.23 to 0.29)	-0.29 (-0.50 to -0.09)	-1.02 (-1.21 to -0.84)	0.17 (0.11 to 0.23)	-0.72 (-0.86 to -0.57)
Slovakia	-0.14 (-0.16 to -0.12)	0.00 (-0.01 to 0.02)	-0.60 (-0.84 to -0.36)	-0.95 (-1.21 to -0.69)	-0.37 (-0.41 to -0.32)	-0.82 (-1.02 to -0.62)
Slovenia	0.07 (0.03 to 0.10)	0.13 (0.08 to 0.18)	-2.62 (-2.90 to -2.33)	-3.25 (-3.57 to -2.93)	-0.09 (-0.19 to 0.00)	-2.53 (-2.77 to -2.28)
**Eastern Europe**	0.04 (0.01 to 0.06)	-0.03 (-0.11 to 0.04)	1.77 (1.24 to 2.31)	2.16 (1.47 to 2.86)	-0.10 (-0.15 to -0.05)	1.76 (1.19 to 2.33)
Belarus	0.03 (0.02 to 0.04)	0.09 (0.06 to 0.11)	1.66 (0.99 to 2.33)	2.01 (1.16 to 2.87)	-0.04 (-0.08 to -0.01)	1.48 (0.83 to 2.13)
Estonia	0.06 (0.05 to 0.07)	0.06 (0.05 to 0.07)	-0.11 (-0.66 to 0.44)	-0.09 (-0.72 to 0.55)	-0.14 (-0.16 to -0.11)	-0.11 (-0.58 to 0.37)
Latvia	0.01 (-0.01 to 0.04)	0.07 (0.02 to 0.12)	0.19 (-0.28 to 0.66)	0.30 (-0.26 to 0.86)	-0.09 (-0.20 to 0.02)	0.19 (-0.24 to 0.62)
Lithuania	0.08 (0.04 to 0.13)	0.11 (0.06 to 0.17)	2.66 (1.94 to 3.38)	2.82 (1.97 to 3.68)	0.15 (0.05 to 0.26)	2.15 (1.48 to 2.82)
Republic of Moldova	-0.03 (-0.04 to -0.01)	-0.03 (-0.04 to -0.01)	-1.20 (-1.58 to -0.82)	-1.27 (-1.68 to -0.87)	-0.36 (-0.39 to -0.33)	-1.17 (-1.53 to -0.81)
Russian Federation	0.04 (0.00 to 0.09)	-0.06 (-0.16 to 0.05)	1.85 (1.27 to 2.43)	2.15 (1.38 to 2.92)	-0.13 (-0.20 to -0.06)	1.75 (1.12 to 2.38)
Ukraine	0.04 (0.04 to 0.05)	0.04 (0.01 to 0.06)	2.07 (1.53 to 2.63)	2.79 (2.10 to 3.49)	0.02 (-0.01 to 0.06)	2.30 (1.73 to 2.88)
**High income**	0.07 (0.05 to 0.09)	-0.08 (-0.12 to -0.04)	-1.30 (-1.35 to -1.24)	-1.51 (-1.58 to -1.45)	-0.37 (-0.43 to -0.30)	-1.20 (-1.27 to -1.13)
**Australasia**	0.14 (0.12 to 0.16)	0.05 (-0.02 to 0.11)	-1.12 (-1.28 to -0.95)	-1.23 (-1.41 to -1.05)	0.02 (-0.03 to 0.07)	-0.79 (-0.93 to -0.66)
Australia	0.12 (0.08 to 0.15)	0.02 (-0.07 to 0.11)	-1.06 (-1.22 to -0.89)	-1.16 (-1.34 to -0.98)	-0.01 (-0.08 to 0.06)	-0.78 (-0.92 to -0.63)
New Zealand	0.25 (0.22 to 0.28)	0.20 (0.18 to 0.22)	-1.48 (-1.64 to -1.31)	-1.68 (-1.84 to -1.52)	0.20 (0.16 to 0.24)	-0.86 (-0.95 to -0.78)
**High-income Asia Pacific**	-0.07 (-0.13 to 0.00)	0.27 (0.18 to 0.36)	-2.74 (-2.91 to -2.57)	-3.31 (-3.45 to -3.17)	-0.09 (-0.14 to -0.04)	-2.28 (-2.42 to -2.15)
Brunei Darussalam	-0.34 (-0.36 to -0.32)	0.00 (-0.01 to 0.00)	-1.85 (-2.09 to -1.62)	-2.18 (-2.46 to -1.89)	-0.44 (-0.49 to -0.38)	-1.73 (-1.96 to -1.50)
Japan	-0.02 (-0.05 to	0.31 (0.22 to	-2.00 (-2.16 to	-2.29 (-2.39 to	0.14 (0.11 to	-1.37 (-1.46 to

	0.01)	0.39)	-1.84)	-2.19)	0.18)	-1.29)
Republic of Korea	-0.28 (-0.45 to -0.10)	0.15 (0.02 to 0.29)	-5.08 (-5.44 to -4.72)	-5.65 (-5.97 to -5.32)	-0.77 (-0.93 to -0.62)	-4.62 (-4.94 to -4.30)
Singapore	-0.15 (-0.16 to -0.14)	0.09 (0.08 to 0.10)	-3.12 (-3.28 to -2.96)	-3.72 (-3.90 to -3.54)	-0.37 (-0.40 to -0.35)	-2.21 (-2.36 to -2.07)
**High-income North America**	-0.02 (-0.08 to 0.05)	-0.53 (-0.64 to -0.41)	-0.17 (-0.21 to -0.13)	-0.09 (-0.14 to -0.04)	-0.67 (-0.83 to -0.51)	-0.25 (-0.32 to -0.17)
Canada	0.07 (0.07 to 0.08)	0.06 (0.05 to 0.08)	-0.97 (-1.02 to -0.91)	-0.89 (-0.96 to -0.81)	-0.07 (-0.12 to -0.02)	-0.62 (-0.68 to -0.55)
Greenland	0.01 (0.01 to 0.02)	-0.03 (-0.04 to -0.02)	-1.48 (-1.64 to -1.33)	-1.63 (-1.73 to -1.52)	-0.29 (-0.31 to -0.26)	-1.42 (-1.51 to -1.32)
United States of America	-0.02 (-0.09 to 0.05)	-0.58 (-0.70 to -0.45)	-0.10 (-0.14 to -0.05)	-0.02 (-0.08 to 0.03)	-0.74 (-0.91 to -0.57)	-0.21 (-0.29 to -0.14)
**Southern Latin America**	0.12 (0.07 to 0.17)	-0.04 (-0.09 to 0.01)	-0.82 (-0.94 to -0.69)	-1.10 (-1.26 to -0.94)	-0.27 (-0.36 to -0.19)	-0.95 (-1.08 to -0.82)
Argentina	0.07 (0.01 to 0.14)	-0.08 (-0.15 to -0.02)	-0.47 (-0.64 to -0.31)	-0.70 (-0.90 to -0.50)	-0.29 (-0.40 to -0.18)	-0.63 (-0.79 to -0.46)
Chile	0.19 (0.15 to 0.22)	0.03 (0.00 to 0.06)	-1.71 (-1.82 to -1.60)	-2.07 (-2.22 to -1.91)	-0.32 (-0.40 to -0.25)	-1.80 (-1.93 to -1.66)
Uruguay	0.25 (0.22 to 0.27)	0.08 (0.07 to 0.08)	-1.04 (-1.10 to -0.99)	-1.41 (-1.47 to -1.34)	-0.05 (-0.06 to -0.04)	-1.11 (-1.17 to -1.05)
**Western Europe**	0.18 (0.15 to 0.21)	0.10 (0.07 to 0.13)	-1.55 (-1.61 to -1.50)	-1.95 (-2.03 to -1.88)	-0.22 (-0.28 to -0.15)	-1.48 (-1.55 to -1.42)
Andorra	0.23 (0.19 to 0.27)	0.06 (0.05 to 0.07)	-0.71 (-0.82 to -0.61)	-0.84 (-0.90 to -0.77)	-0.03 (-0.05 to -0.02)	-0.62 (-0.68 to -0.57)
Austria	0.06 (0.02 to 0.10)	-0.05 (-0.11 to 0.00)	-2.39 (-2.48 to -2.30)	-2.80 (-2.88 to -2.72)	-0.27 (-0.38 to -0.16)	-2.06 (-2.12 to -2.00)
Belgium	0.32 (0.24 to 0.39)	0.19 (0.10 to 0.28)	-1.03 (-1.21 to -0.85)	-1.15 (-1.31 to -0.98)	0.34 (0.17 to 0.51)	-0.78 (-0.93 to -0.63)
Cyprus	0.22 (0.15 to 0.29)	0.14 (0.08 to 0.20)	-2.32 (-2.45 to -2.18)	-2.49 (-2.61 to -2.37)	0.14 (-0.03 to 0.31)	-1.89 (-1.97 to -1.81)
Denmark	0.12 (0.08 to 0.15)	-0.07 (-0.10 to -0.05)	-1.27 (-1.76 to -0.79)	-1.87 (-2.34 to -1.40)	-0.31 (-0.35 to -0.26)	-1.49 (-1.86 to -1.13)
Finland	0.16 (0.08 to 0.24)	-0.07 (-0.14 to 0.00)	-0.32 (-0.57 to -0.07)	0.06 (-0.29 to 0.40)	0.06 (-0.06 to 0.19)	0.06 (-0.22 to 0.34)
France	0.13 (0.06 to 0.20)	-0.02 (-0.17 to 0.13)	-2.23 (-2.30 to -2.15)	-2.45 (-2.53 to -2.37)	-0.24 (-0.37 to -0.11)	-1.98 (-2.04 to -1.92)
Germany	0.27 (0.20 to 0.34)	0.12 (0.01 to 0.24)	-1.39 (-1.48 to -1.29)	-2.04 (-2.19 to -1.90)	0.03 (-0.17 to 0.23)	-1.59 (-1.71 to -1.48)
Greece	0.13 (0.09 to 0.16)	0.06 (0.06 to 0.07)	-1.09 (-1.26 to -0.92)	-0.96 (-1.14 to -0.77)	-0.04 (-0.06 to -0.03)	-0.65 (-0.78 to -0.52)
Iceland	0.44 (0.40 to 0.49)	0.19 (0.14 to 0.23)	-1.74 (-1.83 to -1.64)	-1.94 (-2.02 to -1.85)	0.31 (0.24 to 0.38)	-1.19 (-1.24 to -1.13)
Ireland	0.33 (0.29 to	0.08 (0.08 to	-1.27 (-1.52 to	-1.05 (-1.36 to	-0.06 (-0.08 to	-0.76 (-0.99 to

	0.36)	0.09)	-1.03)	-0.73)	-0.03)	-0.52)
Israel	0.42 (0.39 to 0.46)	0.05 (0.03 to 0.07)	-1.14 (-1.39 to -0.88)	-1.49 (-1.73 to -1.25)	-0.11 (-0.14 to -0.08)	-1.07 (-1.24 to -0.90)
Italy	0.06 (0.04 to 0.09)	0.14 (0.08 to 0.19)	-2.45 (-2.58 to -2.33)	-3.03 (-3.19 to -2.88)	-0.49 (-0.63 to -0.34)	-2.08 (-2.25 to -1.91)
Luxembourg	0.13 (0.05 to 0.22)	-0.01 (-0.13 to 0.11)	-2.16 (-2.27 to -2.04)	-2.57 (-2.67 to -2.48)	-0.16 (-0.37 to 0.05)	-1.98 (-2.08 to -1.88)
Malta	0.36 (0.28 to 0.44)	0.15 (0.11 to 0.19)	-1.82 (-1.92 to -1.71)	-1.79 (-1.87 to -1.71)	0.20 (0.08 to 0.33)	-1.21 (-1.29 to -1.14)
Netherlands	0.25 (0.23 to 0.26)	0.05 (0.04 to 0.05)	-1.47 (-1.76 to -1.19)	-1.80 (-2.11 to -1.49)	-0.38 (-0.45 to -0.31)	-1.42 (-1.66 to -1.18)
Norway	0.21 (0.19 to 0.24)	0.40 (0.29 to 0.52)	-1.47 (-1.56 to -1.37)	-1.88 (-1.99 to -1.77)	-0.02 (-0.05 to 0.01)	-1.01 (-1.06 to -0.96)
Portugal	0.23 (0.18 to 0.27)	0.16 (0.14 to 0.17)	-2.57 (-2.69 to -2.45)	-3.22 (-3.33 to -3.11)	0.17 (0.12 to 0.22)	-2.67 (-2.78 to -2.56)
Monaco	0.26 (0.23 to 0.29)	0.06 (0.06 to 0.06)	-0.08 (-0.20 to 0.04)	-0.26 (-0.35 to -0.18)	0.01 (-0.01 to 0.02)	-0.19 (-0.25 to -0.13)
San Marino	0.25 (0.22 to 0.28)	0.07 (0.07 to 0.07)	-0.37 (-0.48 to -0.26)	-0.53 (-0.65 to -0.41)	0.02 (0.00 to 0.04)	-0.38 (-0.47 to -0.29)
Spain	0.14 (0.11 to 0.18)	0.05 (-0.06 to 0.15)	-2.26 (-2.35 to -2.16)	-2.89 (-3.00 to -2.78)	-0.19 (-0.28 to -0.09)	-2.33 (-2.43 to -2.23)
Sweden	0.35 (0.29 to 0.42)	0.10 (0.01 to 0.19)	-1.01 (-1.08 to -0.94)	-1.23 (-1.33 to -1.14)	-0.01 (-0.09 to 0.08)	-0.79 (-0.85 to -0.73)
Switzerland	0.33 (0.25 to 0.42)	0.15 (0.01 to 0.28)	-0.73 (-1.02 to -0.45)	-1.47 (-1.69 to -1.26)	-0.01 (-0.29 to 0.28)	-1.05 (-1.27 to -0.83)
United Kingdom	0.22 (0.11 to 0.34)	0.25 (0.13 to 0.37)	0.34 (0.13 to 0.54)	0.48 (0.19 to 0.77)	-0.16 (-0.41 to 0.10)	0.26 (0.05 to 0.46)
**Latin America and Caribbean**	-0.01 (-0.02 to 0.00)	0.02 (-0.02 to 0.06)	-1.15 (-1.24 to -1.06)	-1.56 (-1.69 to -1.44)	-0.36 (-0.38 to -0.33)	-1.32 (-1.42 to -1.22)
**Andean Latin America**	0.05 (0.04 to 0.06)	-0.21 (-0.27 to -0.16)	-1.59 (-1.70 to -1.47)	-2.80 (-3.04 to -2.55)	-0.62 (-0.68 to -0.56)	-2.45 (-2.66 to -2.23)
Ecuador	0.09 (0.07 to 0.12)	0.07 (0.05 to 0.08)	-0.40 (-0.64 to -0.16)	-1.31 (-1.54 to -1.08)	-0.24 (-0.27 to -0.21)	-1.13 (-1.32 to -0.94)
Peru	0.01 (-0.01 to 0.02)	-0.38 (-0.47 to -0.29)	-2.37 (-2.57 to -2.16)	-3.82 (-4.21 to -3.43)	-0.82 (-0.91 to -0.73)	-3.31 (-3.65 to -2.96)
Bolivia (Plurinational State of)	0.02 (0.02 to 0.03)	-0.03 (-0.05 to -0.01)	-1.02 (-1.08 to -0.95)	-1.91 (-2.01 to -1.82)	-0.51 (-0.54 to -0.48)	-1.73 (-1.83 to -1.64)
**Caribbean**	0.10 (0.10 to 0.11)	0.07 (0.07 to 0.08)	-1.08 (-1.29 to -0.86)	-1.21 (-1.47 to -0.95)	-0.05 (-0.06 to -0.03)	-0.98 (-1.19 to -0.76)
Antigua and Barbuda	0.09 (0.09 to 0.10)	0.10 (0.09 to 0.10)	-0.94 (-1.19 to -0.69)	-1.26 (-1.48 to -1.03)	0.02 (0.00 to 0.04)	-0.91 (-1.08 to -0.74)
Barbados	0.09 (0.09 to 0.10)	0.09 (0.08 to 0.09)	-0.94 (-1.05 to -0.82)	-1.27 (-1.41 to -1.13)	-0.02 (-0.04 to 0.00)	-0.92 (-1.02 to -0.81)

Belize	0.16 (0.11 to 0.21)	0.09 (0.09 to 0.10)	-0.22 (-0.56 to 0.13)	-0.29 (-0.59 to 0.00)	0.03 (0.01 to 0.05)	-0.23 (-0.46 to 0.01)
Bermuda	0.09 (0.08 to 0.09)	0.14 (0.13 to 0.14)	-2.84 (-3.09 to -2.58)	-2.98 (-3.27 to -2.69)	-0.03 (-0.06 to -0.01)	-1.97 (-2.19 to -1.74)
Bahamas	0.07 (0.06 to 0.08)	0.06 (0.05 to 0.06)	-1.25 (-1.40 to -1.09)	-1.38 (-1.56 to -1.20)	-0.12 (-0.14 to -0.11)	-1.11 (-1.26 to -0.96)
Cuba	0.15 (0.14 to 0.15)	0.08 (0.07 to 0.09)	-0.62 (-0.88 to -0.37)	-0.54 (-0.80 to -0.29)	-0.02 (-0.05 to 0.01)	-0.38 (-0.56 to -0.21)
Dominica	0.07 (0.06 to 0.09)	0.02 (0.02 to 0.03)	-0.92 (-1.01 to -0.83)	-1.03 (-1.11 to -0.94)	-0.06 (-0.09 to -0.04)	-0.80 (-0.87 to -0.73)
Dominican Republic	0.17 (0.16 to 0.18)	0.10 (0.08 to 0.11)	-0.97 (-1.38 to -0.56)	-1.49 (-2.00 to -0.98)	-0.06 (-0.10 to -0.02)	-1.24 (-1.66 to -0.82)
Grenada	0.10 (0.09 to 0.10)	0.07 (0.06 to 0.08)	-0.98 (-1.15 to -0.81)	-1.20 (-1.39 to -1.01)	-0.05 (-0.09 to -0.01)	-0.96 (-1.12 to -0.80)
Guyana	0.09 (0.08 to 0.09)	0.05 (0.04 to 0.06)	-0.84 (-1.06 to -0.61)	-0.81 (-1.00 to -0.62)	-0.18 (-0.22 to -0.15)	-0.73 (-0.90 to -0.56)
Haiti	-0.06 (-0.07 to -0.05)	0.09 (0.09 to 0.10)	-1.26 (-1.37 to -1.14)	-1.47 (-1.63 to -1.31)	-0.19 (-0.20 to -0.18)	-1.30 (-1.44 to -1.15)
Jamaica	0.20 (0.18 to 0.22)	0.09 (0.08 to 0.09)	-0.85 (-1.16 to -0.53)	-1.32 (-1.69 to -0.95)	0.07 (0.04 to 0.09)	-0.86 (-1.10 to -0.61)
Puerto Rico	0.16 (0.14 to 0.18)	0.10 (0.10 to 0.11)	-2.78 (-3.06 to -2.50)	-3.11 (-3.42 to -2.80)	-0.11 (-0.14 to -0.08)	-2.42 (-2.67 to -2.17)
Saint Kitts and Nevis	0.02 (0.01 to 0.03)	0.03 (0.03 to 0.04)	-1.88 (-2.16 to -1.60)	-2.31 (-2.64 to -1.99)	-0.29 (-0.35 to -0.23)	-1.93 (-2.21 to -1.66)
Saint Lucia	0.11 (0.10 to 0.11)	0.09 (0.08 to 0.09)	-1.98 (-2.34 to -1.62)	-1.99 (-2.33 to -1.65)	-0.06 (-0.10 to -0.03)	-1.55 (-1.83 to -1.28)
Saint Vincent and the Grenadines	0.15 (0.14 to 0.16)	0.05 (0.05 to 0.06)	-0.82 (-1.12 to -0.52)	-1.00 (-1.27 to -0.74)	-0.05 (-0.07 to -0.04)	-0.79 (-0.99 to -0.58)
Suriname	0.12 (0.11 to 0.12)	0.07 (0.06 to 0.08)	-0.98 (-1.18 to -0.79)	-1.32 (-1.48 to -1.15)	-0.17 (-0.18 to -0.15)	-1.12 (-1.25 to -0.98)
Trinidad and Tobago	0.11 (0.10 to 0.11)	0.03 (0.02 to 0.05)	-1.70 (-1.91 to -1.49)	-1.76 (-1.97 to -1.56)	-0.23 (-0.27 to -0.19)	-1.38 (-1.53 to -1.22)
United States Virgin Islands	0.17 (0.15 to 0.19)	0.10 (0.09 to 0.11)	-0.32 (-0.51 to -0.12)	-0.63 (-0.79 to -0.47)	0.00 (-0.01 to 0.02)	-0.49 (-0.61 to -0.36)
**Central Latin America**	0.06 (0.05 to 0.07)	0.09 (0.07 to 0.11)	-1.17 (-1.28 to -1.05)	-1.44 (-1.59 to -1.28)	-0.31 (-0.38 to -0.25)	-1.22 (-1.36 to -1.09)
Venezuela (Bolivarian Republic of)	0.04 (0.03 to 0.05)	0.01 (0.00 to 0.01)	-1.09 (-1.27 to -0.91)	-1.29 (-1.50 to -1.09)	-0.35 (-0.39 to -0.30)	-1.04 (-1.20 to -0.88)
Colombia	-0.10 (-0.12 to -0.09)	0.09 (0.09 to 0.10)	-1.68 (-1.83 to -1.53)	-1.96 (-2.09 to -1.82)	-0.32 (-0.35 to -0.28)	-1.41 (-1.51 to -1.32)
Costa Rica	0.01 (0.00 to 0.02)	0.04 (0.02 to 0.05)	-0.56 (-0.83 to -0.29)	-0.73 (-0.99 to -0.47)	-0.17 (-0.21 to -0.13)	-0.58 (-0.77 to -0.39)
El Salvador	0.01 (0.00 to	-0.09 (-0.13 to	-1.83 (-2.13 to	-2.28 (-2.65 to	-0.61 (-0.70 to	-1.96 (-2.28 to

	0.01)	-0.05)	-1.52)	-1.91)	-0.51)	-1.64)
Guatemala	-0.08 (-0.09 to -0.06)	-0.10 (-0.15 to -0.04)	-1.44 (-1.61 to -1.27)	-1.87 (-2.08 to -1.67)	-0.72 (-0.79 to -0.65)	-1.73 (-1.92 to -1.55)
Honduras	-0.03 (-0.04 to -0.02)	0.02 (0.01 to 0.04)	0.14 (-0.02 to 0.29)	-1.09 (-1.16 to -1.01)	-0.44 (-0.47 to -0.41)	-1.01 (-1.08 to -0.93)
Mexico	0.13 (0.11 to 0.16)	0.14 (0.11 to 0.18)	-1.14 (-1.29 to -0.99)	-1.37 (-1.54 to -1.21)	-0.25 (-0.34 to -0.16)	-1.19 (-1.33 to -1.04)
Nicaragua	-0.08 (-0.10 to -0.06)	0.08 (0.06 to 0.09)	0.25 (0.08 to 0.42)	-0.17 (-0.30 to -0.04)	-0.35 (-0.40 to -0.30)	-0.21 (-0.31 to -0.10)
Panama	0.09 (0.08 to 0.11)	-0.03 (-0.05 to -0.01)	-0.75 (-0.90 to -0.60)	-0.90 (-1.14 to -0.65)	-0.31 (-0.36 to -0.26)	-0.71 (-0.89 to -0.54)
**Tropical Latin America**	-0.12 (-0.15 to -0.09)	-0.03 (-0.09 to 0.04)	-1.08 (-1.14 to -1.02)	-1.41 (-1.46 to -1.37)	-0.46 (-0.53 to -0.39)	-1.20 (-1.23 to -1.17)
Brazil	-0.12 (-0.15 to -0.09)	-0.02 (-0.09 to 0.04)	-1.10 (-1.16 to -1.05)	-1.44 (-1.48 to -1.40)	-0.46 (-0.54 to -0.39)	-1.22 (-1.24 to -1.19)
Paraguay	-0.06 (-0.06 to -0.05)	-0.10 (-0.12 to -0.08)	0.29 (0.21 to 0.38)	-0.08 (-0.22 to 0.07)	-0.40 (-0.46 to -0.34)	-0.17 (-0.28 to -0.06)
**North Africa and Middle East**	0.05 (0.04 to 0.06)	0.15 (0.14 to 0.15)	-1.50 (-1.57 to -1.42)	-1.62 (-1.67 to -1.56)	-0.01 (-0.03 to 0.01)	-1.37 (-1.42 to -1.32)
Afghanistan	-0.09 (-0.10 to -0.07)	0.06 (0.05 to 0.07)	-1.18 (-1.35 to -1.01)	-1.40 (-1.61 to -1.20)	-0.09 (-0.12 to -0.05)	-1.26 (-1.44 to -1.07)
Algeria	0.15 (0.13 to 0.18)	0.08 (0.08 to 0.09)	-1.86 (-1.93 to -1.80)	-2.14 (-2.20 to -2.08)	-0.10 (-0.12 to -0.08)	-1.69 (-1.75 to -1.64)
Bahrain	0.10 (0.08 to 0.13)	0.06 (0.05 to 0.07)	-2.71 (-3.02 to -2.40)	-3.15 (-3.44 to -2.86)	-0.29 (-0.33 to -0.26)	-2.69 (-2.93 to -2.45)
Egypt	-0.06 (-0.09 to -0.03)	0.10 (0.09 to 0.11)	-0.78 (-0.92 to -0.65)	-0.90 (-1.03 to -0.78)	-0.14 (-0.18 to -0.11)	-0.86 (-0.97 to -0.74)
Iraq	0.03 (-0.02 to 0.08)	0.12 (0.11 to 0.14)	-1.62 (-1.75 to -1.50)	-2.05 (-2.17 to -1.92)	-0.03 (-0.05 to 0.00)	-1.57 (-1.66 to -1.48)
Iran (Islamic Republic of)	0.16 (0.07 to 0.24)	0.12 (0.09 to 0.15)	-1.53 (-1.69 to -1.36)	-1.74 (-1.89 to -1.59)	-0.05 (-0.10 to 0.01)	-1.28 (-1.39 to -1.17)
Jordan	-0.11 (-0.13 to -0.08)	0.13 (0.13 to 0.14)	-2.65 (-2.89 to -2.41)	-2.85 (-3.12 to -2.57)	0.11 (0.08 to 0.13)	-2.14 (-2.34 to -1.95)
Kuwait	0.22 (0.20 to 0.24)	0.16 (0.14 to 0.18)	-0.19 (-0.64 to 0.27)	-0.68 (-1.17 to -0.18)	0.06 (0.03 to 0.09)	-0.42 (-0.75 to -0.09)
Lebanon	0.11 (0.08 to 0.13)	0.20 (0.19 to 0.20)	-1.35 (-1.44 to -1.25)	-1.46 (-1.60 to -1.33)	0.07 (0.05 to 0.09)	-1.06 (-1.17 to -0.96)
Libya	0.09 (0.07 to 0.10)	0.12 (0.11 to 0.12)	-1.40 (-1.53 to -1.27)	-1.60 (-1.73 to -1.47)	-0.05 (-0.07 to -0.02)	-1.28 (-1.38 to -1.17)
Morocco	0.14 (0.11 to 0.18)	0.08 (0.07 to 0.09)	-1.13 (-1.30 to -0.96)	-1.44 (-1.52 to -1.36)	-0.14 (-0.17 to -0.12)	-1.19 (-1.26 to -1.12)
Palestine	-0.07 (-0.11 to -0.04)	0.07 (0.05 to 0.09)	-1.17 (-1.30 to -1.03)	-1.41 (-1.56 to -1.27)	-0.04 (-0.08 to 0.01)	-1.12 (-1.25 to -0.99)
Oman	0.22 (0.18 to	0.07 (0.05 to	-0.73 (-0.98 to	-1.19 (-1.44 to	-0.18 (-0.20 to	-0.97 (-1.17 to

	0.26)	0.09)	-0.49)	-0.93)	-0.15)	-0.78)
Qatar	0.18 (0.16 to 0.20)	0.15 (0.14 to 0.16)	-0.03 (-0.27 to 0.22)	-1.01 (-1.20 to -0.81)	0.00 (-0.02 to 0.01)	-0.85 (-1.01 to -0.68)
Saudi Arabia	0.02 (-0.02 to 0.06)	0.12 (0.12 to 0.13)	-2.28 (-2.38 to -2.19)	-2.61 (-2.72 to -2.50)	-0.15 (-0.16 to -0.13)	-2.25 (-2.35 to -2.15)
Sudan	-0.18 (-0.21 to -0.16)	0.10 (0.09 to 0.11)	-1.04 (-1.09 to -0.98)	-1.34 (-1.41 to -1.28)	-0.03 (-0.05 to -0.01)	-1.15 (-1.20 to -1.10)
Syrian Arab Republic	-0.11 (-0.14 to -0.09)	0.13 (0.13 to 0.14)	-1.24 (-1.47 to -1.01)	-1.58 (-1.84 to -1.32)	-0.02 (-0.05 to 0.01)	-1.27 (-1.48 to -1.05)
Tunisia	0.07 (0.03 to 0.11)	0.11 (0.10 to 0.11)	-1.05 (-1.08 to -1.01)	-1.34 (-1.38 to -1.30)	-0.06 (-0.09 to -0.03)	-1.03 (-1.06 to -0.99)
Turkey	0.12 (0.10 to 0.15)	0.37 (0.33 to 0.40)	-1.21 (-1.34 to -1.09)	-2.19 (-2.26 to -2.12)	0.38 (0.30 to 0.46)	-1.43 (-1.49 to -1.36)
United Arab Emirates	0.29 (0.26 to 0.32)	0.06 (0.04 to 0.07)	-1.56 (-1.99 to -1.12)	-1.51 (-1.85 to -1.17)	-0.09 (-0.12 to -0.06)	-1.19 (-1.45 to -0.94)
Yemen	-0.31 (-0.33 to -0.29)	0.08 (0.08 to 0.09)	-1.37 (-1.46 to -1.27)	-1.51 (-1.61 to -1.42)	-0.05 (-0.07 to -0.02)	-1.29 (-1.37 to -1.21)
**South Asia**	0.09 (0.06 to 0.13)	0.14 (0.11 to 0.17)	-1.94 (-2.11 to -1.78)	-1.92 (-2.06 to -1.78)	-0.15 (-0.23 to -0.07)	-1.68 (-1.79 to -1.57)
Bangladesh	-0.04 (-0.09 to 0.00)	0.03 (-0.05 to 0.11)	-3.61 (-3.83 to -3.39)	-3.86 (-4.01 to -3.71)	-0.57 (-0.69 to -0.45)	-3.42 (-3.55 to -3.29)
Bhutan	-0.11 (-0.12 to -0.10)	0.17 (0.13 to 0.21)	-1.39 (-1.44 to -1.34)	-1.88 (-1.94 to -1.81)	-0.40 (-0.44 to -0.36)	-1.70 (-1.76 to -1.65)
India	0.11 (0.06 to 0.15)	0.18 (0.14 to 0.22)	-1.90 (-2.08 to -1.73)	-1.84 (-2.00 to -1.68)	-0.06 (-0.16 to 0.03)	-1.60 (-1.73 to -1.46)
Nepal	0.24 (0.20 to 0.27)	-0.13 (-0.16 to -0.10)	-1.44 (-1.62 to -1.26)	-1.94 (-2.13 to -1.74)	-0.38 (-0.41 to -0.35)	-1.75 (-1.93 to -1.57)
Pakistan	0.08 (0.05 to 0.12)	0.00 (-0.03 to 0.03)	-0.90 (-1.01 to -0.79)	-0.90 (-1.02 to -0.78)	-0.50 (-0.57 to -0.44)	-0.86 (-0.96 to -0.75)
**Southeast Asia, east Asia, and Oceania**	-0.43 (-0.54 to -0.32)	0.02 (-0.04 to 0.09)	-2.52 (-2.59 to -2.44)	-2.83 (-2.89 to -2.76)	-0.82 (-0.90 to -0.74)	-2.48 (-2.54 to -2.43)
**East Asia**	-0.55 (-0.69 to -0.41)	-0.01 (-0.09 to 0.07)	-3.31 (-3.45 to -3.17)	-3.89 (-4.01 to -3.77)	-0.87 (-0.96 to -0.79)	-3.19 (-3.27 to -3.11)
China	-0.56 (-0.70 to -0.42)	-0.01 (-0.08 to 0.07)	-3.38 (-3.53 to -3.24)	-3.99 (-4.12 to -3.87)	-0.89 (-0.97 to -0.80)	-3.26 (-3.34 to -3.17)
Democratic People's Republic of Korea	-0.56 (-0.62 to -0.51)	-0.21 (-0.24 to -0.18)	-1.01 (-1.11 to -0.92)	-1.21 (-1.26 to -1.16)	-0.35 (-0.38 to -0.32)	-1.09 (-1.13 to -1.05)
Taiwan (Province of China)	-0.25 (-0.28 to -0.22)	-0.17 (-0.19 to -0.15)	-2.86 (-3.10 to -2.62)	-2.63 (-2.85 to -2.41)	-0.52 (-0.55 to -0.49)	-2.37 (-2.57 to -2.18)
**Oceania**	-0.30 (-0.33 to -0.27)	0.03 (0.01 to 0.04)	-0.87 (-0.91 to -0.83)	-0.86 (-0.89 to -0.83)	-0.03 (-0.06 to 0.00)	-0.76 (-0.79 to -0.73)
American Samoa	0.06 (-0.03 to	0.02 (0.01 to	-0.93 (-1.09 to	-0.92 (-1.08 to	-0.06 (-0.11 to	-0.81 (-0.96 to

	0.15)	0.03)	-0.78)	-0.76)	-0.01)	-0.67)
Cook Islands	0.09 (0.03 to 0.15)	0.09 (0.08 to 0.10)	-1.85 (-2.00 to -1.70)	-1.89 (-2.07 to -1.70)	0.03 (-0.01 to 0.06)	-1.60 (-1.76 to -1.43)
Micronesia (Federated States of)	-0.17 (-0.23 to -0.11)	-0.01 (-0.03 to 0.01)	-1.77 (-1.99 to -1.55)	-1.84 (-2.05 to -1.63)	-0.42 (-0.49 to -0.36)	-1.73 (-1.93 to -1.54)
Fiji	-0.08 (-0.10 to -0.06)	0.02 (0.00 to 0.04)	-0.79 (-0.89 to -0.69)	-0.88 (-0.98 to -0.78)	0.00 (-0.04 to 0.05)	-0.76 (-0.86 to -0.67)
Guam	0.24 (0.22 to 0.25)	-0.03 (-0.05 to -0.01)	-1.68 (-2.00 to -1.36)	-1.10 (-1.32 to -0.88)	-0.17 (-0.24 to -0.10)	-0.98 (-1.17 to -0.78)
Kiribati	-0.18 (-0.25 to -0.10)	-0.02 (-0.03 to -0.01)	-1.59 (-1.68 to -1.51)	-1.75 (-1.83 to -1.66)	-0.25 (-0.29 to -0.21)	-1.66 (-1.75 to -1.58)
Marshall Islands	-0.10 (-0.14 to -0.06)	-0.03 (-0.05 to -0.01)	-1.59 (-1.64 to -1.54)	-1.59 (-1.63 to -1.54)	-0.30 (-0.33 to -0.28)	-1.49 (-1.53 to -1.45)
Northern Mariana Islands	0.12 (0.06 to 0.19)	0.07 (0.05 to 0.09)	-1.62 (-1.74 to -1.50)	-1.75 (-1.88 to -1.61)	-0.07 (-0.11 to -0.04)	-1.60 (-1.73 to -1.48)
Papua New Guinea	-0.36 (-0.39 to -0.33)	0.04 (0.02 to 0.06)	-0.46 (-0.50 to -0.42)	-0.56 (-0.60 to -0.51)	0.00 (-0.04 to 0.03)	-0.48 (-0.52 to -0.45)
Nauru	-0.20 (-0.24 to -0.15)	0.05 (0.02 to 0.07)	-1.11 (-1.33 to -0.88)	-1.19 (-1.44 to -0.95)	-0.05 (-0.09 to -0.02)	-1.12 (-1.34 to -0.89)
Niue	-0.02 (-0.06 to 0.03)	0.06 (0.05 to 0.07)	-1.46 (-1.52 to -1.40)	-1.68 (-1.75 to -1.60)	-0.07 (-0.09 to -0.04)	-1.52 (-1.59 to -1.45)
Palau	0.00 (-0.06 to 0.06)	0.04 (0.04 to 0.05)	-0.91 (-0.98 to -0.84)	-0.85 (-0.93 to -0.78)	-0.05 (-0.07 to -0.03)	-0.76 (-0.83 to -0.69)
Samoa	-0.09 (-0.16 to -0.03)	0.02 (0.01 to 0.03)	-1.19 (-1.33 to -1.05)	-1.23 (-1.35 to -1.11)	-0.10 (-0.12 to -0.07)	-1.12 (-1.23 to -1.01)
Solomon Islands	-0.42 (-0.47 to -0.36)	0.01 (-0.02 to 0.03)	-0.91 (-0.95 to -0.87)	-0.92 (-0.96 to -0.87)	-0.21 (-0.25 to -0.18)	-0.87 (-0.91 to -0.83)
Tokelau	0.14 (0.10 to 0.18)	0.04 (0.02 to 0.06)	-1.52 (-1.58 to -1.45)	-1.63 (-1.69 to -1.57)	-0.18 (-0.22 to -0.14)	-1.48 (-1.54 to -1.42)
Tonga	-0.32 (-0.41 to -0.23)	-0.03 (-0.05 to -0.02)	-1.19 (-1.30 to -1.08)	-1.18 (-1.27 to -1.08)	-0.28 (-0.32 to -0.24)	-1.10 (-1.19 to -1.01)
Tuvalu	-0.10 (-0.14 to -0.05)	0.01 (0.00 to 0.02)	-1.49 (-1.62 to -1.36)	-1.57 (-1.71 to -1.43)	-0.30 (-0.35 to -0.24)	-1.47 (-1.60 to -1.34)
Vanuatu	-0.21 (-0.23 to -0.19)	-0.05 (-0.07 to -0.03)	-1.35 (-1.49 to -1.21)	-1.33 (-1.50 to -1.17)	-0.29 (-0.33 to -0.25)	-1.24 (-1.40 to -1.09)
**Southeast Asia**	-0.14 (-0.16 to -0.13)	0.12 (0.10 to 0.13)	-1.37 (-1.41 to -1.33)	-1.73 (-1.77 to -1.68)	-0.48 (-0.53 to -0.43)	-1.62 (-1.66 to -1.58)
Cambodia	-0.42 (-0.45 to -0.38)	0.06 (0.04 to 0.07)	-1.44 (-1.53 to -1.36)	-1.84 (-1.94 to -1.74)	-0.55 (-0.60 to -0.50)	-1.78 (-1.88 to -1.68)
Indonesia	0.03 (0.00 to 0.05)	0.06 (0.03 to 0.08)	-0.54 (-0.61 to -0.47)	-1.31 (-1.38 to -1.23)	-0.86 (-0.94 to -0.78)	-1.27 (-1.34 to -1.20)
Lao Peoples's Democratic	-0.38 (-0.42 to -0.34)	0.08 (0.08 to 0.09)	-2.04 (-2.14 to -1.93)	-2.34 (-2.44 to -2.25)	-0.42 (-0.46 to -0.38)	-2.20 (-2.30 to -2.11)

Republic						
Malaysia	0.23 (0.20 to 0.25)	0.21 (0.19 to 0.23)	-0.61 (-1.07 to -0.16)	-0.81 (-1.11 to -0.52)	-0.03 (-0.05 to -0.01)	-0.72 (-0.98 to -0.45)
Maldives	0.03 (-0.02 to 0.08)	0.31 (0.30 to 0.33)	-3.08 (-3.31 to -2.84)	-3.60 (-3.91 to -3.30)	-0.08 (-0.15 to -0.02)	-2.95 (-3.22 to -2.67)
Mauritius	0.00 (-0.02 to 0.02)	0.06 (0.05 to 0.07)	-2.88 (-3.15 to -2.62)	-3.07 (-3.37 to -2.77)	-0.26 (-0.29 to -0.22)	-2.78 (-3.04 to -2.51)
Myanmar	0.02 (0.01 to 0.04)	0.05 (0.04 to 0.07)	-1.82 (-1.91 to -1.73)	-1.96 (-2.04 to -1.87)	-0.49 (-0.54 to -0.43)	-1.87 (-1.95 to -1.79)
Philippines	-0.46 (-0.49 to -0.44)	0.12 (0.08 to 0.16)	-2.08 (-2.24 to -1.91)	-1.89 (-2.04 to -1.74)	-0.12 (-0.20 to -0.03)	-1.70 (-1.85 to -1.56)
Sri Lanka	0.03 (0.00 to 0.07)	0.16 (0.13 to 0.19)	-2.53 (-3.02 to -2.04)	-3.15 (-3.74 to -2.56)	-0.29 (-0.35 to -0.22)	-2.79 (-3.30 to -2.27)
Seychelles	0.03 (0.02 to 0.05)	0.08 (0.06 to 0.10)	-1.08 (-1.29 to -0.87)	-1.37 (-1.58 to -1.16)	-0.29 (-0.32 to -0.26)	-1.27 (-1.47 to -1.08)
Viet Nam	-0.59 (-0.62 to -0.57)	0.26 (0.25 to 0.27)	-2.32 (-2.67 to -1.97)	-2.35 (-2.73 to -1.97)	-0.05 (-0.11 to 0.01)	-2.11 (-2.45 to -1.76)
Thailand	-0.08 (-0.12 to -0.05)	0.11 (0.10 to 0.12)	-1.32 (-1.42 to -1.21)	-1.47 (-1.59 to -1.34)	-0.24 (-0.31 to -0.17)	-1.33 (-1.45 to -1.21)
Timor-Leste	-0.28 (-0.31 to -0.24)	0.18 (0.16 to 0.19)	-0.92 (-1.05 to -0.79)	-1.31 (-1.50 to -1.12)	-0.19 (-0.23 to -0.15)	-1.21 (-1.39 to -1.04)
**Sub-Saharan Africa**	-0.29 (-0.33 to -0.25)	0.09 (0.08 to 0.10)	-0.86 (-0.94 to -0.78)	-1.02 (-1.10 to -0.95)	0.03 (0.01 to 0.05)	-0.94 (-1.00 to -0.87)
**Central sub-Saharan Africa**	-0.18 (-0.22 to -0.14)	0.18 (0.17 to 0.19)	-1.04 (-1.10 to -0.98)	-1.12 (-1.18 to -1.06)	0.30 (0.28 to 0.33)	-0.99 (-1.05 to -0.94)
Angola	-0.44 (-0.48 to -0.40)	0.20 (0.19 to 0.21)	-1.00 (-1.07 to -0.93)	-1.25 (-1.33 to -1.16)	0.17 (0.15 to 0.19)	-1.14 (-1.22 to -1.06)
Central African Republic	-0.22 (-0.25 to -0.20)	0.08 (0.07 to 0.09)	-0.54 (-0.61 to -0.47)	-0.69 (-0.75 to -0.63)	0.12 (0.11 to 0.14)	-0.63 (-0.68 to -0.57)
Congo	-0.42 (-0.45 to -0.40)	0.15 (0.13 to 0.17)	-1.24 (-1.38 to -1.10)	-1.58 (-1.72 to -1.43)	0.09 (0.05 to 0.13)	-1.44 (-1.57 to -1.31)
Democratic Republic of the Congo	-0.10 (-0.15 to -0.05)	0.19 (0.18 to 0.20)	-1.08 (-1.15 to -1.01)	-1.06 (-1.14 to -0.99)	0.39 (0.36 to 0.43)	-0.93 (-0.99 to -0.87)
Equatorial Guinea	0.06 (0.03 to 0.10)	0.18 (0.15 to 0.22)	-2.46 (-2.66 to -2.25)	-3.09 (-3.34 to -2.85)	-0.17 (-0.20 to -0.14)	-2.77 (-3.00 to -2.55)
Gabon	-0.27 (-0.30 to -0.24)	0.11 (0.10 to 0.13)	-1.17 (-1.31 to -1.02)	-1.49 (-1.62 to -1.35)	-0.02 (-0.06 to 0.01)	-1.36 (-1.48 to -1.23)
**Eastern sub-Saharan Africa**	-0.22 (-0.25 to -0.19)	0.10 (0.09 to 0.12)	-0.94 (-1.01 to -0.88)	-1.15 (-1.21 to -1.09)	0.06 (0.02 to 0.09)	-1.06 (-1.11 to -1.01)
Burundi	-0.17 (-0.20 to -0.15)	0.18 (0.17 to 0.20)	-1.22 (-1.36 to -1.08)	-1.37 (-1.50 to -1.23)	0.42 (0.34 to 0.49)	-1.23 (-1.36 to -1.11)

Comoros	-0.12 (-0.14 to -0.09)	0.11 (0.10 to 0.11)	-0.81 (-0.97 to -0.66)	-1.00 (-1.23 to -0.77)	-0.01 (-0.02 to 0.01)	-0.91 (-1.11 to -0.70)
Djibouti	-0.06 (-0.07 to -0.04)	0.09 (0.07 to 0.11)	-0.73 (-0.81 to -0.64)	-0.88 (-1.01 to -0.76)	-0.08 (-0.12 to -0.04)	-0.81 (-0.92 to -0.70)
Eritrea	-0.29 (-0.32 to -0.27)	0.15 (0.14 to 0.16)	0.04 (-0.14 to 0.21)	-0.17 (-0.34 to -0.01)	0.18 (0.16 to 0.21)	-0.15 (-0.30 to 0.01)
Ethiopia	-0.16 (-0.18 to -0.13)	0.04 (0.03 to 0.05)	-1.58 (-1.70 to -1.46)	-2.05 (-2.18 to -1.93)	-0.21 (-0.24 to -0.19)	-1.94 (-2.05 to -1.82)
Kenya	0.01 (0.00 to 0.03)	0.06 (0.04 to 0.09)	-0.04 (-0.18 to 0.10)	-0.06 (-0.24 to 0.13)	0.06 (-0.02 to 0.14)	-0.05 (-0.22 to 0.12)
Madagascar	-0.23 (-0.26 to -0.21)	0.13 (0.12 to 0.15)	-0.58 (-0.65 to -0.51)	-0.79 (-0.82 to -0.75)	0.30 (0.24 to 0.36)	-0.69 (-0.72 to -0.66)
Malawi	-0.23 (-0.29 to -0.16)	0.17 (0.16 to 0.18)	-1.27 (-1.46 to -1.08)	-1.43 (-1.64 to -1.22)	0.27 (0.24 to 0.31)	-1.31 (-1.50 to -1.11)
Mozambique	-0.55 (-0.61 to -0.49)	0.17 (0.16 to 0.19)	0.12 (0.04 to 0.21)	0.27 (0.13 to 0.40)	0.22 (0.16 to 0.28)	0.26 (0.14 to 0.38)
Rwanda	-0.34 (-0.39 to -0.30)	0.16 (0.15 to 0.18)	-2.78 (-3.11 to -2.45)	-3.02 (-3.35 to -2.69)	0.04 (0.01 to 0.07)	-2.80 (-3.11 to -2.49)
Somalia	-0.08 (-0.10 to -0.06)	0.09 (0.08 to 0.10)	-0.41 (-0.52 to -0.30)	-0.47 (-0.57 to -0.38)	0.11 (0.09 to 0.13)	-0.43 (-0.52 to -0.34)
South Sudan	0.03 (0.01 to 0.05)	0.10 (0.08 to 0.11)	-0.28 (-0.34 to -0.23)	-0.48 (-0.55 to -0.42)	0.13 (0.10 to 0.15)	-0.43 (-0.49 to -0.37)
United Republic of Tanzania	-0.20 (-0.22 to -0.17)	0.12 (0.11 to 0.14)	-0.72 (-0.98 to -0.46)	-0.78 (-1.03 to -0.52)	0.16 (0.13 to 0.19)	-0.69 (-0.92 to -0.46)
Uganda	-0.56 (-0.61 to -0.51)	0.11 (0.10 to 0.13)	-1.04 (-1.23 to -0.85)	-1.11 (-1.34 to -0.88)	0.07 (0.05 to 0.10)	-1.00 (-1.22 to -0.79)
Zambia	-0.34 (-0.39 to -0.29)	0.10 (0.09 to 0.12)	-1.14 (-1.38 to -0.89)	-1.14 (-1.39 to -0.89)	-0.06 (-0.09 to -0.03)	-1.07 (-1.31 to -0.84)
**Southern sub-Saharan Africa**	-0.20 (-0.21 to -0.19)	0.06 (0.04 to 0.08)	-0.77 (-1.11 to -0.44)	-1.09 (-1.48 to -0.70)	-0.18 (-0.19 to -0.17)	-0.95 (-1.29 to -0.62)
Botswana	-0.25 (-0.27 to -0.23)	0.01 (-0.02 to 0.04)	-1.59 (-2.03 to -1.16)	-1.67 (-2.12 to -1.22)	-0.41 (-0.51 to -0.31)	-1.54 (-1.95 to -1.12)
Lesotho	-0.29 (-0.33 to -0.25)	-0.01 (-0.04 to 0.02)	0.46 (0.23 to 0.68)	0.51 (0.28 to 0.74)	-0.25 (-0.28 to -0.21)	0.42 (0.22 to 0.62)
Namibia	-0.31 (-0.36 to -0.27)	0.11 (0.09 to 0.14)	-0.97 (-1.19 to -0.75)	-1.06 (-1.29 to -0.82)	-0.18 (-0.20 to -0.15)	-0.95 (-1.16 to -0.74)
South Africa	-0.12 (-0.13 to -0.10)	0.07 (0.06 to 0.09)	-0.96 (-1.37 to -0.54)	-1.54 (-2.05 to -1.03)	-0.22 (-0.26 to -0.18)	-1.32 (-1.76 to -0.89)
Eswatini	-0.40 (-0.48 to -0.32)	0.03 (0.00 to 0.06)	-0.13 (-0.56 to 0.31)	-0.11 (-0.55 to 0.33)	-0.15 (-0.19 to -0.12)	-0.11 (-0.51 to 0.28)
Zimbabwe	-0.40 (-0.43 to -0.38)	-0.02 (-0.08 to 0.05)	0.13 (-0.04 to 0.30)	0.30 (0.12 to 0.48)	0.02 (-0.10 to 0.15)	0.27 (0.12 to 0.42)
**Western**	-0.43 (-0.48 to	0.07 (0.06 to	-0.82 (-0.92 to	-0.95 (-1.02 to	0.01 (-0.02 to	-0.87 (-0.94 to

**sub-Saharan Africa**	-0.37)	0.08)	-0.73)	-0.87)	0.04)	-0.81)
Benin	-0.40 (-0.46 to -0.33)	0.06 (0.05 to 0.08)	-1.07 (-1.18 to -0.95)	-1.11 (-1.23 to -0.99)	0.02 (-0.02 to 0.07)	-1.02 (-1.12 to -0.91)
Burkina Faso	-0.19 (-0.26 to -0.11)	0.12 (0.11 to 0.13)	-1.15 (-1.27 to -1.03)	-1.00 (-1.15 to -0.85)	0.32 (0.28 to 0.36)	-0.88 (-1.02 to -0.75)
Cameroon	-0.29 (-0.30 to -0.28)	0.07 (0.05 to 0.08)	-1.50 (-1.71 to -1.29)	-1.42 (-1.63 to -1.21)	-0.01 (-0.04 to 0.03)	-1.30 (-1.49 to -1.10)
Chad	-0.17 (-0.20 to -0.13)	0.03 (0.02 to 0.03)	-0.27 (-0.42 to -0.12)	-0.27 (-0.43 to -0.12)	-0.03 (-0.07 to 0.01)	-0.26 (-0.40 to -0.11)
Ghana	-0.61 (-0.68 to -0.54)	0.11 (0.10 to 0.13)	-1.47 (-1.54 to -1.41)	-1.43 (-1.49 to -1.36)	0.10 (0.05 to 0.14)	-1.31 (-1.37 to -1.25)
Guinea	-0.32 (-0.35 to -0.29)	0.05 (0.04 to 0.06)	-0.56 (-0.68 to -0.43)	-0.59 (-0.69 to -0.49)	-0.06 (-0.09 to -0.03)	-0.55 (-0.64 to -0.45)
Guinea-Bissau	-0.40 (-0.44 to -0.35)	0.02 (0.01 to 0.03)	-0.62 (-0.68 to -0.56)	-0.72 (-0.80 to -0.64)	-0.21 (-0.23 to -0.19)	-0.69 (-0.76 to -0.61)
Liberia	-0.24 (-0.29 to -0.19)	0.11 (0.10 to 0.12)	-1.20 (-1.31 to -1.08)	-1.38 (-1.51 to -1.26)	0.03 (-0.01 to 0.06)	-1.28 (-1.40 to -1.17)
Mali	-0.06 (-0.08 to -0.05)	0.10 (0.09 to 0.12)	-1.39 (-1.47 to -1.30)	-1.48 (-1.57 to -1.39)	0.27 (0.17 to 0.36)	-1.34 (-1.42 to -1.25)
Mauritania	-0.46 (-0.51 to -0.41)	0.08 (0.07 to 0.09)	-2.40 (-2.49 to -2.31)	-2.53 (-2.61 to -2.44)	-0.08 (-0.11 to -0.04)	-2.31 (-2.38 to -2.23)
Niger	-0.34 (-0.38 to -0.30)	0.07 (0.04 to 0.09)	-0.76 (-0.84 to -0.67)	-0.96 (-1.07 to -0.86)	0.00 (-0.09 to 0.09)	-0.88 (-0.98 to -0.78)
Nigeria	-0.52 (-0.60 to -0.45)	0.07 (0.06 to 0.08)	-0.48 (-0.65 to -0.32)	-0.72 (-0.85 to -0.59)	-0.08 (-0.09 to -0.06)	-0.67 (-0.79 to -0.55)
Cape Verde	-0.31 (-0.35 to -0.28)	0.14 (0.12 to 0.16)	-1.13 (-1.38 to -0.89)	-1.42 (-1.59 to -1.26)	0.05 (0.01 to 0.09)	-1.24 (-1.38 to -1.09)
Côte d'Ivoire	-0.51 (-0.54 to -0.48)	0.06 (0.05 to 0.08)	-1.27 (-1.48 to -1.06)	-1.33 (-1.54 to -1.11)	0.02 (-0.01 to 0.06)	-1.21 (-1.40 to -1.02)
The Gambia	-0.33 (-0.35 to -0.32)	0.00 (-0.01 to 0.01)	-1.34 (-1.54 to -1.14)	-1.45 (-1.68 to -1.21)	-0.26 (-0.29 to -0.23)	-1.34 (-1.55 to -1.12)
Sao Tome and Principe	-0.43 (-0.47 to -0.39)	0.06 (0.05 to 0.07)	-1.51 (-1.84 to -1.18)	-1.75 (-2.10 to -1.40)	-0.19 (-0.21 to -0.17)	-1.62 (-1.94 to -1.30)
Senegal	-0.31 (-0.36 to -0.26)	0.06 (0.05 to 0.07)	-1.03 (-1.15 to -0.91)	-1.10 (-1.26 to -0.94)	-0.02 (-0.05 to 0.01)	-1.00 (-1.14 to -0.85)
Sierra Leone	-0.54 (-0.62 to -0.47)	0.16 (0.14 to 0.18)	-1.15 (-1.22 to -1.08)	-1.12 (-1.17 to -1.08)	0.54 (0.47 to 0.61)	-0.98 (-1.02 to -0.94)
Togo	-0.18 (-0.21 to -0.14)	0.11 (0.09 to 0.13)	-0.92 (-1.00 to -0.85)	-0.95 (-1.03 to -0.87)	0.25 (0.19 to 0.31)	-0.85 (-0.91 to -0.78)

Note: North Africa & Middle East and South Asia are both super-regions and regions. CI=confidence interval. DALY=disability-adjusted life-year. EAPC=estimated annual percentage change. YLD=year lived with disability. YLL=year of life lost.

Supplementary Table 5 Disability-adjusted life-years (DALYs), with 95% uncertainty intervals, due to digestive diseases in 2019 for both sexes by SDI quintile and location

**Characteristics**	**Cirrhosis and other chronic liver diseases**	**Upper digestive system diseases**	**Gallbladder and biliary diseases**	**Inguinal, femoral, and abdominal hernia**	**Inflammatory bowel disease**	**Pancreatitis**	**Appendicitis**	**Paralytic ileus and intestinal obstruction**	**Vascular intestinal disorders**	**Other digestive diseases**
**Global**	46189416 (43027109-49551292)	14872827 (11337465-20175275)	6352738 (4874475-8250379)	3354016 (2610569-4247142)	1622498 (1356296-1915042)	3641106 (3282953-4026948)	1501492 (1236232-1705756)	7083934 (5648476-8253785)	1872339 (1722038-2034447)	2501402 (2124794-2799241)
**SDI quintile**										
High	4702397 (4508829-4882152)	1484408 (1024785-2210344)	1195591 (912611-1566520)	341845 (261098-432826)	603330 (489511-729627)	389933 (360178-429354)	65652 (53398-80562)	661697 (535884-725012)	519308 (474724-551923)	560689 (429792-716735)
High-middle	7639449 (7168729-8166771)	2560429 (1890809-3616203)	1480358 (1096026-1978931)	533108 (396999-691160)	352794 (285252-432623)	1057815 (962196-1156821)	109306 (92726-132526)	689070 (592690-752968)	583263 (537823-621728)	444203 (323392-513363)
Middle	14074007 (12898464-15406240)	4141729 (3122989-5692454)	2058323 (1558009-2715934)	945356 (729102-1207538)	291088 (240412-345833)	813217 (727040-960673)	317880 (265061-369324)	1535402 (1290025-1789571)	334507 (307149-363488)	655952 (553484-753382)
Low-middle	12974479 (11742205-14369549)	4430754 (3516359-5816397)	1136297 (876795-1478050)	875136 (679692-1122028)	236584 (189248-283804)	919233 (751489-1066252)	614999 (495566-709957)	2245752 (1673992-2674023)	287609 (232710-360988)	411357 (338670-476136)
Low	6773037 (5866959-7846211)	2266582 (1795714-2931535)	479136 (376300-594544)	655949 (499451-832433)	137867 (100264-180825)	459380 (367334-583539)	392572 (284827-505022)	1948061 (1417088-2494504)	146732 (116415-185308)	427420 (301664-550111)
**GBD regions**										

Andean Latin America	373058 (294137-465085)	140863 (97847-207886)	50543 (40737-62083)	52197 (38838-70120)	5942 (4666-7395)	43655 (34042-57110)	24903 (19692-31923)	72359 (53952-95137)	10477 (8623-12696)	28269 (20685-36782)
Australasia	63253 (58694-67742)	38198 (24715-59523)	22701 (17212-29842)	9583 (7277-12328)	13328 (10488-16560)	7137 (6313-8100)	1641 (1257-2143)	14204 (10721-16172)	10346 (8968-11527)	15825 (12605-22994)
Caribbean	278278 (219587-341080)	129127 (93267-178869)	33874 (26528-42644)	44267 (31469-60163)	8900 (6523-11709)	20255 (16880-24620)	19774 (14156-27771)	46752 (34290-66086)	12529 (10427-15182)	25116 (19118-32921)
Central Asia	1173145 (1054258-1309149)	168376 (127011-232484)	56602 (41414-76057)	39478 (28590-53646)	20237 (16357-24806)	60311 (51432-69088)	9684 (7864-12463)	44880 (37020-54092)	14238 (12816-15824)	29001 (22672-35084)
Central Europe	964512 (839764-1091671)	298870 (224717-413799)	168292 (122481-228933)	52616 (40955-67152)	54694 (42729-67570)	140578 (123451-158896)	9252 (7565-12887)	77394 (64147-90277)	80015 (70998-89933)	49464 (34233-59160)
Central Latin America	2031944 (1740913-2343602)	546521 (381813-805680)	393301 (297340-517353)	185912 (144104-237198)	36178 (30219-43014)	141733 (121886-165140)	79612 (63718-97580)	211165 (163583-258318)	91301 (78161-104954)	156372 (112990-188854)
Central Sub-Saharan Africa	843049 (629422-1106950)	261369 (196003-345968)	53722 (36371-71194)	89254 (62543-132401)	15574 (10733-22385)	35823 (21455-62657)	43183 (24388-69515)	183618 (111141-304070)	21400 (13134-33494)	79128 (38817-111913)
East Asia	4698614 (3982096-5513225)	2433506 (1929172-3160818)	1709612 (1186854-2408010)	300521 (207472-407260)	239963 (187123-299808)	319973 (255865-381414)	68712 (55191-84652)	415868 (356362-483170)	130067 (106734-149275)	300069 (252684-358011)
Eastern Europe	2575335 (2298785-2874695)	611398 (477392-812140)	219514 (165062-293549)	121328 (89360-159944)	77123 (63287-94008)	558129 (481179-640436)	21213 (17341-30074)	99240 (82580-120044)	263459 (236239-291640)	113938 (64668-142092)

Eastern Sub-Saharan Africa	2608125 (2191259-3151159)	720077 (556341-942914)	178794 (128881-269562)	204924 (150651-290015)	52928 (39435-74044)	103187 (65328-170639)	124840 (74786-195219)	687833 (516400-885431)	54424 (40217-66452)	205919 (107462-301135)
High-income Asia Pacific	776896 (717258-824271)	234503 (165283-346646)	328527 (246786-436576)	83668 (58698-112642)	78601 (55261-105049)	48064 (41966-56862)	12236 (9472-15548)	147817 (110992-168027)	58402 (50095-63696)	74928 (53353-88658)
High-income North America	1955333 (1892680-2014259)	526571 (345756-803432)	339838 (259903-439670)	92204 (74022-113485)	245234 (200564-294018)	145495 (134413-161035)	22691 (18436-27444)	222558 (185287-244896)	211893 (196390-223239)	237188 (199681-326057)
North Africa and Middle East	2878426 (2126424-3565004)	930743 (618724-1423304)	280263 (214922-355906)	111488 (80063-150494)	72755 (56928-91112)	91833 (74033-111078)	70903 (51605-87877)	256131 (205760-327623)	48945 (42093-56445)	100126 (74657-119155)
Oceania	45978 (35985-57692)	24737 (19742-30771)	7220 (5196-10247)	4515 (3129-6640)	1696 (1107-2576)	3181 (2194-4459)	1674 (940-2395)	7061 (4071-11328)	842 (641-1096)	2922 (2145-3857)
South Asia	12436281 (10954317-14414136)	4489057 (3397445-6123397)	849893 (616184-1146403)	919587 (700868-1191111)	221978 (166881-278494)	909994 (707700-1093716)	703819 (557425-836936)	2248080 (1572772-2689131)	298900 (217378-406713)	147492 (119799-175502)
Southeast Asia	5915349 (5207219-6642923)	950173 (751560-1238851)	459557 (359409-560923)	348090 (279868-421610)	46686 (38847-55186)	254592 (204677-376979)	102875 (75572-121780)	694738 (571637-810106)	73414 (64359-83289)	217369 (182709-256581)
Southern Latin America	372368 (351788-394659)	117490 (75982-182613)	48157 (40596-59304)	32935 (25229-42278)	7740 (6670-9152)	40292 (36540-45859)	8121 (6663-10668)	65454 (52808-73052)	31543 (28747-34701)	33474 (19151-40516)
Southern Sub-Saharan Africa	310794 (272944-353505)	184063 (144959-244635)	40601 (34324-47042)	51209 (37842-68707)	9230 (7542-10926)	21221 (16850-25384)	14079 (11730-17255)	105011 (87812-125276)	9973 (8797-11181)	41094 (34998-46944)

Tropical Latin America	1193340 (1134408-1261300)	501792 (337756-759628)	347165 (271905-445754)	159147 (123400-203160)	46997 (40115-54972)	175860 (155755-189220)	49542 (38112-58203)	181150 (151258-202384)	91117 (84334-98288)	134342 (98881-172427)
Western Europe	1832992 (1752144-1930380)	676124 (471853-997888)	577768 (443914-754038)	193550 (148284-247107)	310691 (255911-371106)	199700 (183864-224327)	28489 (23147-37211)	336898 (275956-381593)	299807 (271457-323892)	266616 (183532-325687)
Western Sub-Saharan Africa	2862345 (2196426-3688872)	889269 (648853-1199212)	186794 (122670-250866)	257542 (190011-332759)	56026 (35327-78051)	320090 (226679-458546)	84249 (53617-120589)	965723 (628048-1439964)	59247 (44506-79067)	242752 (168297-346344)

Abbreviations: GBD, Global Burden of Diseases, Injuries, and Risk Factors Study; SDI, Socio-demographic Index.

Supplementary Table 6 Percentage contribution of risk factors to all-age disability-adjusted life-years (DALYs) of digestive diseases in 2019 for both sexes, globally and by regions

Location	Smoking	Alcohol use	Drug use	High body-mass index
**Global**	1.68%	26.93%	7.71%	2.30%
Andean Latin America	0.50%	26.61%	2.62%	2.86%
Australasia	1.34%	25.95%	12.85%	5.71%
Caribbean	1.38%	27.53%	2.34%	2.06%
Central Asia	1.38%	41.71%	11.86%	1.58%
Central Europe	2.79%	41.12%	7.91%	4.47%
Central Latin America	0.82%	33.22%	3.81%	4.50%
Central Sub-Saharan Africa	0.60%	22.04%	1.07%	0.62%
East Asia	3.91%	28.87%	11.26%	3.68%
Eastern Europe	2.69%	45.75%	9.93%	2.40%
Eastern Sub-Saharan Africa	0.60%	23.74%	3.59%	0.76%
High-income Asia Pacific	2.28%	28.82%	6.38%	3.29%
High-income North America	1.44%	35.78%	20.32%	4.34%
North Africa and Middle East	1.27%	7.21%	4.33%	2.77%
Oceania	3.42%	14.13%	10.09%	1.95%
South Asia	1.24%	21.84%	9.18%	0.89%
Southeast Asia	1.57%	27.23%	7.58%	1.32%
Southern Latin America	1.35%	38.64%	15.52%	2.64%
Southern Sub-Saharan Africa	1.77%	25.59%	5.61%	2.29%
Tropical Latin America	1.55%	28.66%	2.26%	5.58%
Western Europe	2.04%	31.38%	5.47%	5.01%
Western Sub-Saharan Africa	0.50%	23.58%	0.07%	0.57%
